# Country-specific citation disparities in Naunyn–Schmiedeberg’s Archives of Pharmacology from 2001 to 2024

**DOI:** 10.1007/s00210-025-04499-9

**Published:** 2025-08-14

**Authors:** Roland Seifert, Waseem Hassan

**Affiliations:** 1 Institute of Pharmacology, Hannover Medical School, Carl-Neuberg-Str. 1, Hannover, 30625 Germany; 2 Institute of Chemical Sciences, University of Peshawar, Peshawar, Khyber Pakhtunkhwa 25120 Pakistan

**Keywords:** Scopus, NSAP, Bibliometric analysis, Publications, Citations, Disparity

## Abstract

This bibliometric study examined disparities in research output and citation impact in 4155 (including all types of documents) research documents published in *Naunyn–Schmiedeberg’s Archives of Pharmacology* (NSAP) from 2001 to 2024. The objective was to assess how publication volume and citations vary across countries, i.e., high-income countries (HICs), upper-middle-income and lower-middle-income countries (UMICs and LMICs). Germany led in both number of publications (770 publications) and total citations (17,569), followed by China, India, the United States, and Egypt. Regression-based citation analysis by country group revealed pronounced disparities. In the low-output category (11–51 publications), HICs such as Norway, Switzerland, and Sweden demonstrated citation performance far above expectations, while several LMICs underperformed despite comparable output levels. In the moderate-output group (85–169 publications), citation correlation weakened overall. While the Netherlands and the UK maintained high impact, middle-income countries like South Korea and Saudi Arabia showed limited citation impact. Among high-output countries (240–769 publications), citation performance was more consistent (*R*^2^ = 0.731), with Germany, the US and Japan significantly outperforming other contributors. Conversely, UMICs such as China, India, Iran, and Egypt—despite high output—lagged in citation returns, suggesting a gap between quantity and influence. The disparities in citation performance persisted in both original and review categories, underscoring the structural imbalance in global research influence. In conclusion, while NSAP has expanded its international scope and output, substantial citation inequality remains across country income groups. These findings highlight the need for targeted editorial, collaborative, and policy strategies to enhance research visibility and recognition in UMICs and LMICs.

**Supplementary Information:**

The online version contains supplementary material available at 10.1007/s00210-025-04499-9.

## Introduction

In recent years, our group has undertaken several focused studies examining the structural framework, thematic developments, and ethical practices associated with *Naunyn–Schmiedeberg’s Archives of Pharmacology* (NSAP). Specifically, we tried to contribute to a deeper understanding of the journal’s evolution, challenges, and impact. This includes a comprehensive bibliometric analysis outlining contemporary research trends and scholarly contributions (Dats et al., 2023), as well as a historical investigation of publication and citation dynamics between 1947 and 1974 (Basol and Seifert 2024). Reflecting on the journal’s 150-year history, we have also assessed its editorial trajectory and future directions (Hattori and Seifert 2023).

In a recent series of studies, our group conducted a comprehensive bibliometric examination of NSAP. Hassan et al. (2025) analyzed 25,931 documents published between 1873 and 2025, challenging the perception of NSAP as a predominantly German journal by highlighting increasing international participation from 104 countries. Notably, Germany’s leading role (based on number of publications) has declined in recent years, with China, India, and Iran emerging as major contributors.

Building on this, Abdelwahab et al. (2025a) compared Germany’s pharmacology output in NSAP to other venues, revealing that between 2019 and 2024, only 192 German articles appeared in NSAP, compared to 16,775 published elsewhere—reflecting a shift driven by impact-focused publishing practices.

In a related study, Abdelwahab et al. (2025b) performed the first focused analysis of German Research Foundation (Deutsche Forschungsgemeinschaft (DFG))-sponsored research in NSAP. Using Scopus data from 1969 onward, the study found that only a small fraction of DFG-funded pharmacology-related publications appeared in NSAP—averaging fewer than ten annually between 2015 and 2023.

Abdelwahab et al. (2025c) also conducted detailed authorship analysis of 13,422 NSAP publications from 1969 to 2024. Based on 13,422 publications and over 250,000 citations, the study presented detailed performance metrics for the most prolific authors using multiple bibliometric tools and indicators (e.g., h-index, g-index, Q2 index, and HG composite an index that combines both the h-index and g-index).

Our work has additionally focused on upholding scientific integrity, and reinforcing the importance of trust in scholarly publishing (Sabel and Seifert 2021). These efforts are complemented by the journal’s active measures against unethical authorship practices, including gender-based editorial analysis (Zehetbauer et al. 2022) and retraction of manipulated articles (Seifert, 2021).

We have recently reported an in-depth analysis of post-publication image irregularities, which led to a significant number of retractions and demonstrated the effectiveness of image integrity screening (van Diest et al. 2025). As artificial intelligence continues to influence academic writing, we have also emphasized the importance of transparency by introducing mandatory declarations regarding the use of AI tools and paper mills in review submissions (Seifert et al. 2025).

In parallel, we have also examined structural inequities in scientific recognition, including award disparities in German medical societies including the German Society for Experimental and Clinical Pharmacology and Toxicology (Halling et al. 2025; Steinert and Seifert, 2025), bibliometric comparison of Nobel laureates (Bünemann and Seifert 2024), and underrepresentation in public science platforms such as Biospektrum (Zöllner and Seifert 2024). Furthermore, we have critically assessed the limitations of popular bibliometric indicators, within academic communities (Fox and Seifert 2024).

Building on this foundation, the present study addresses a novel and previously unreported dimension of NSAP: regional disparities in citation of scholarly output. While prior analyses have explored the journal’s thematic evolution, citation patterns, and editorial policies, no systematic investigation has been conducted to examine the geographic distribution of citations of its published research.

By analyzing the regional distribution of contributions to NSAP over time, we aim to uncover underlying patterns of participation, highlight areas of underrepresentation, and trace shifts in the visibility of pharmacological research across different parts of the world. Understanding who contributes to a journal—and who is missing—is vital for assessing the inclusivity of scholarly communication and the accessibility of publication platforms for researchers in low- and middle-income regions.

### Materials and methods

To explore the publication and citation dynamics and regional distribution of contributions to NSAP, a comprehensive bibliometric analysis was conducted using data retrieved from the Scopus database. The analysis spanned a 24-year period, from January 2001 to December 2024, covering the recent evolution of pharmacological research disseminated through NSAP.

The complete metadata for all NSAP publications indexed in Scopus during the study period was downloaded in CSV format. This metadata included key information such as the following:

1. Publication year
2. Document type (e.g., original article, review, editorial, letter, note)
3. Author names and institutional affiliations
4. Country of origin
5. Citation counts

To ensure a comprehensive understanding of NSAP’s publication characteristics, three distinct datasets were analyzed:

1. All document types published in NSAP, including original research articles, reviews, editorials, letters, and notes.
2. A subset focusing exclusively on original research articles, which often represent the core scientific contributions.
3. A separate subset of review articles, which typically synthesize knowledge and are influential within academic discourse.

By examining these three distinct data sets, we were able to capture a comprehensive overview of NSAP’s publishing and citation activity, distinguish between primary knowledge generation (original articles) and knowledge synthesis (reviews), and assess broader editorial patterns, including contributions such as letters and notes. This layered approach enhanced the resolution of our findings and facilitated more targeted insights into the journal’s publishing behavior.

VOSviewer (version 1.6.20) was used to visualize the global distribution of country contributions, with emphasis on identifying geographic patterns.

We identified countries that had published at least 100 documents during the study period—considering both original research articles and reviews. For these countries, annual publication trends were charted to evaluate the evolution and fluctuations in contribution volume.

By focusing on countries with substantial publication output, we were able to trace the growth, stability, or decline of national contributions to NSAP. This approach offered a longitudinal perspective, enabling us to detect emerging contributors, regional shifts in research leadership, and potential saturation or withdrawal trends.

We evaluated the overall regional distribution of authorship to assess whether NSAP maintained a diverse international representation or exhibited concentration from specific regions.

This analysis provided insights into the global inclusivity of NSAP and its appeal or accessibility to researchers worldwide.

For each of the three datasets (all document types, original articles, and reviews), we determined the following:

1. The number of countries involved
2. The total number of publications per country
3. The citation impact (total citations received by each country)

Countries were grouped into regions using standard geographical classifications aligned with those used by the United Nations and World Bank. The regional categories included the following:

1. Europe,
2. North America,
3. Latin America
4. Africa,
5. Middle East
6. Asia,
7. Australia and New Zealand.

To examine disparities based on national wealth, countries were also categorized into income groups using the World Bank’s income classification system (as of the analysis year). The four standard categories used were the following:

1. Low-income,
2. Lower-middle-income,
3. High-income countries.

This dual analysis allowed us to compare quantity with quality, identifying not only the most productive countries but also those whose work generated significant academic attention and impact. Such comparative insight is critical for evaluating research influence, not just output.

The objective extended beyond identifying productive nations to also exploring regional disparities in research contributions to NSAP. This included evaluating the distribution of contributions across continents and regions, and understanding whether NSAP facilitated global participation or remained concentrated in a few geographical areas.

To the best of our knowledge, this is the first study to systematically present the regional distribution of publications and citations of papers published in NSAP. It offers a benchmark for future studies, promotes transparency, and may guide editorial policy to improve global inclusivity.

We performed a simple linear correlation analysis (Pearson’s r) to evaluate the relationship between the number of publications and total citations per country. This analysis was conducted using Microsoft Excel. The goal was to assess the overall strength and direction of the linear association between research output and citation impact. We did not apply non-linear models or non-parametric correlation tests (e.g., Spearman’s rho) in this analysis, as our focus was to determine the general linear trend across countries. No transformations or adjustments for skewed data were applied.

This statistical method enabled us to quantify publication trends and determine significance of growth patterns. Such modeling may help contextualize the journal’s global influence and responsiveness to international research dynamics.

## Results

### The total research output of NSAP

From 2001 to December 2024, NSAP published a total of 4155 documents indexed in the Scopus database. Among these, 3386 were original research articles, making up the majority of the journal’s output. Additionally, there were 505 review articles, 113 errata, 62 conference papers, 42 editorials, 36 retracted publications, 7 letters, 2 notes, and 1 short survey. This data was retrieved on April 20, 2025. This distribution reflected NSAP’s primary focus on original research, with a substantial number of review articles also contributing to the journal’s content.

The annual publication volume showed moderate variation over the two-decade period. Between 2001 and 2019, the journal published between 79 and 163 papers per year. The lowest output during this time was recorded in 2007, with 79 documents, while the highest was in 2001, with 163 publications. Between 2020 and 2022, there was a modest increase, with 229 publications in 2020 and 223 in 2021, followed by a decline to 125 in 2022.

A significant surge in publication output was observed in the most recent years. In 2023, the journal published 305 documents, and in 2024, the number increased sharply to 909.

It is not entirely clear why the number of submissions (and accordingly publications) rose so sharply in 2024. It is likely a mixture of several factors. First, the journal puts emphasis on rigorous but fair peer review, with reasonable revision requests. Second, the journal has a diligent editorial board, rendering fast decisions. Third, the journal has opened its thematic scope substantially during the past years. Fourth, the journal has rigorous policies regarding the fight against paper mills and retractions of fake papers. Fifth, the journal has an efficient editorial manager system, and the publisher Springer Nature provides excellent publication service. Sixth, the journal is available globally and has experienced a surge of downloads, rendering it known globally. Seventh and perhaps most importantly, the editors try their very best to be objective to any given submission, avoiding bias in favor or against certain countries. As a result, authors seem to appreciate the service provided by NSAP as a venue to publish their best research.

**Fig. 1:**
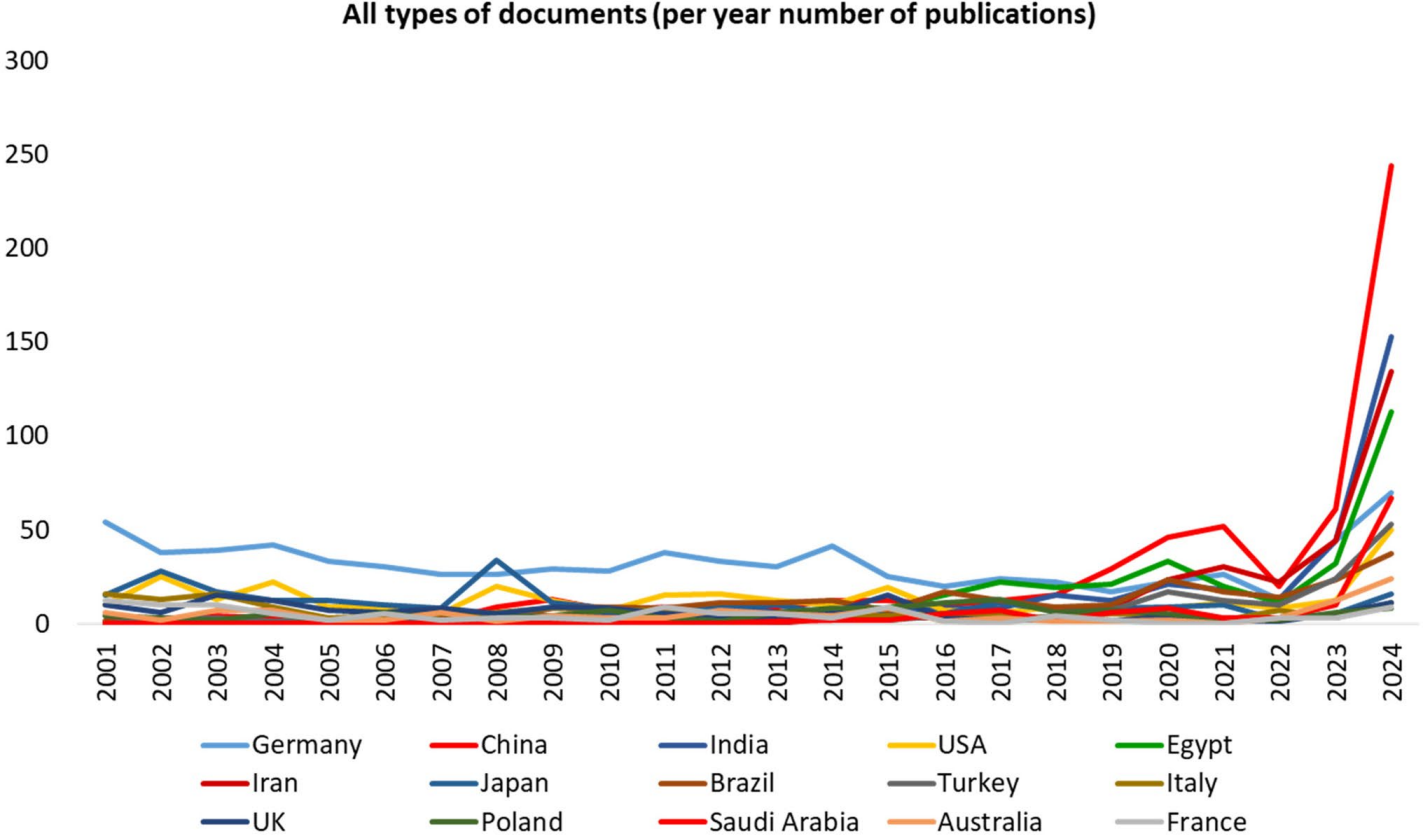
Annual number of publications (all document types) from countries with at least 100 publications in Naunyn–Schmiedeberg’s Archives of Pharmacology (NSAP), 2001–2024

**Fig. 2:**
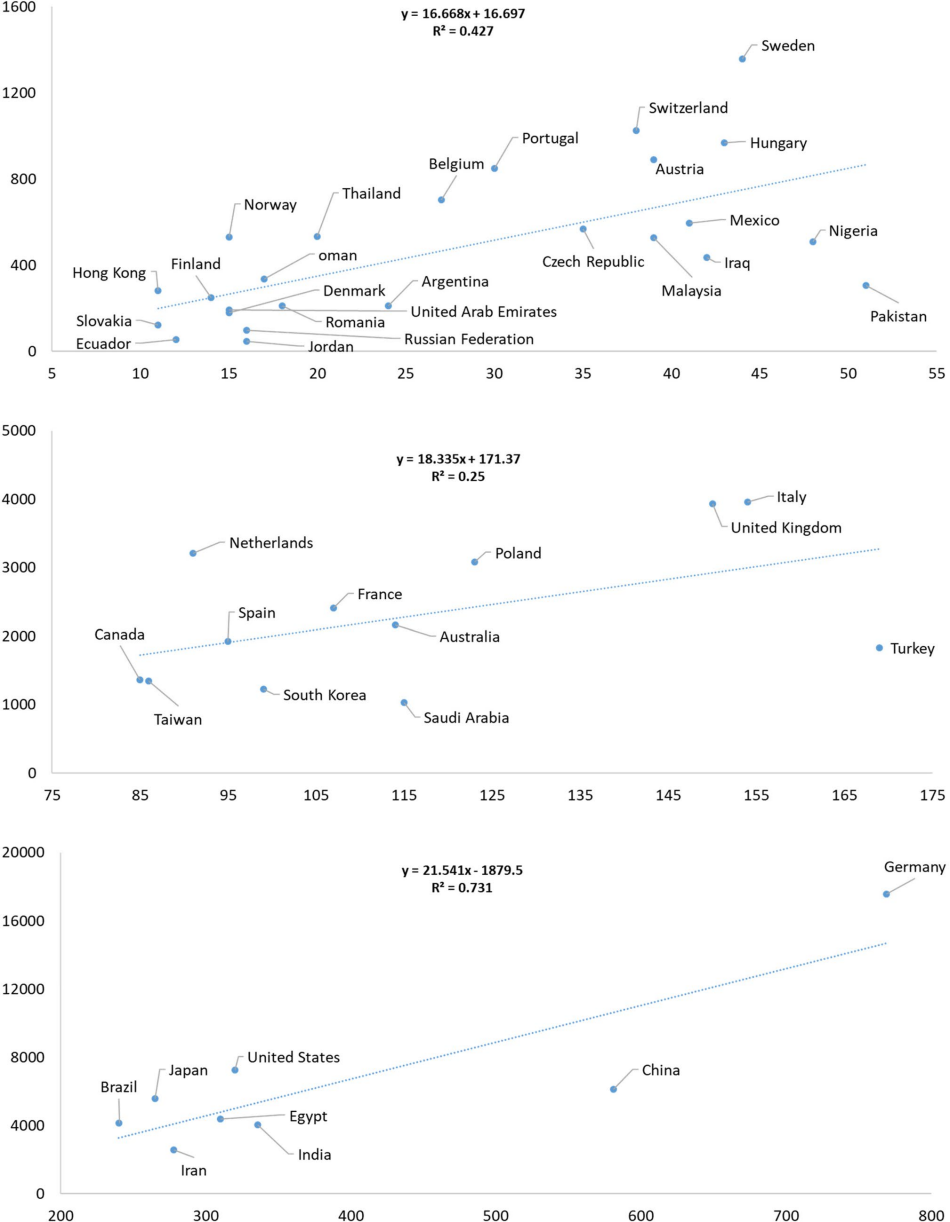
Linear regression analysis of countries, with the number of publications on the x-axis and total citations on the y-axis. The data includes all document types published in Naunyn–Schmiedeberg’s Archives of Pharmacology (NSAP) from 2001 to 2024

The per year number of all types of publications is presented in Supplementary Table 1. A list of countries with at least 100 publications with yearly dynamics is presented in Fig. 1. Notably, a total of 95 countries contributed to all publications. Detailed data on the number of publications and total citations for each country is provided in Supplementary Table 2. The publication output and citation impact varied considerably across countries and were grouped by productivity tiers: 25 countries produced between 11 and 51 publications, 12 countries between 85 and 169 publications, and 8 countries between 240 and 769 publications. This information is visualized in Fig. 2. Regression data for countries with fewer than 10 publications is shown in Supplementary Fig. 1.

#### Low publication volume group (R^2^ = 0.427)

Among countries with relatively low output (11–51 documents), a moderate correlation (R^2^ = 0.427) was observed between publication count and citations. Nine countries stood above the regression line, indicating a citation impact that exceeded expectations based on their modest publication volume. These countries were predominantly high-income countries (HICs) or upper-middle-income countries (UMICs), including Hong Kong (11 documents, 281 citations), Norway (15, 529), Thailand (20, 532), Belgium (27, 702), Portugal (30, 849), Switzerland (38, 1024), Austria (39, 889), Sweden (44, 1356), and Hungary (43, 967). Their performance highlights efficient research dissemination and possibly strong international collaboration or topic relevance despite lower productivity.

Conversely, thirteen countries fell below the regression line, reflecting lower-than-expected citation impact relative to publication volume. This group included several lower-middle-income countries (LMICs) and some HICs, such as Slovakia (11, 122), Ecuador (12, 55), Romania (18, 210), Russian Federation (16, 99), Jordan (16, 47), Argentina (24, 211), Czech Republic (35, 568), Malaysia (39, 527), Mexico (41, 595), Iraq (42, 436), Nigeria (48, 508), and Pakistan (51, 305). Their lower citation returns may indicate challenges such as limited research visibility, less impactful topics, or reduced global collaboration.

#### Mid-Tier publication group (R^2^ = 0.25)

For countries with a moderate publication volume (85–169 documents), the correlation between output and citation impact weakened (*R*^2^ = 0.25), reflecting greater variability in citation numbers. Among these, five countries performed above the regression line, suggesting higher citation influence than their publication counts alone would predict. These were primarily HICs and UMICs: Netherlands (91 documents, 3213 citations); France (107, 2411); Poland (123, 3083); and the UK (150, 3934). Spain (95, 1930) and Australia (114, 2166) were near the regression line, indicating a proportional relationship between output and impact.

In contrast, South Korea (99, 1225); Saudi Arabia (115, 1034); and Turkey (169, 1836)—mostly UMICs—performed below the regression line, indicating citation impact lagging behind their publication output. This disparity might reflect structural or qualitative differences in research impact or international visibility.

#### High publication volume group (R^2^ = 0.731)

Among the most productive countries (240–769 documents), the correlation between output and citation impact was stronger (*R*^2^ = 0.731), indicating more consistent citation performance. Notably, Germany (769 documents, 17,569 citations); the United States (320, 7266); and Japan (265, 5589)—all HICs—were well above the regression line, reflecting exceptionally high citation impact relative to output. This suggests these countries benefit from strong research infrastructure, institutional prestige, and global influence.

Meanwhile, several LMICs and UMICs with high publication volumes—Brazil (240, 4160 citations); Iran (278, 2560); Egypt (310, 4396); India (336, 4051); and China (581, 6128)—fell below the regression line. Despite large output, these countries exhibited comparatively lower citation returns, highlighting potential challenges in research quality, visibility, or citation practices that might limit global influence.

### The total research articles in NSAP

Between 2001 and 2024, NSAP published a total of 3386 original research articles. These publications represented contributions from 88 different countries, reflecting the journal’s international reach and influence in the field of pharmacology.

The annual publication volume showed notable fluctuations over the 24-year period. In the early 2000 s, the journal maintained a relatively stable output, publishing between 123 and 160 articles per year from 2001 to 2004. However, a significant decline was observed between 2005 and 2007, reaching a low of just 64 articles in 2007, which marked the period of lowest productivity during the study timeframe.

Following this decline, a gradual recovery was noted. Publication numbers slowly increased again, reaching over 100 articles per year by 2009. From 2009 to 2019, NSAP maintained a moderate and stable output, typically publishing between 93 and 122 articles annually, indicating a phase of relative consistency in journal activity.

A remarkable change occurred beginning in 2020. In that year, the number of published articles rose sharply to 204, nearly doubling compared to previous years. This upward trend continued with 173 articles in 2021 and a drop to 86 articles in 2022. However, the journal’s publication volume surged again in 2023 with 217 articles, and most strikingly, peaked dramatically in 2024 with an unprecedented 665 articles.

Overall, the data reveal three distinct phases in NSAP’s article publication history: an initial period of moderate activity (2001–2004), a decline and stabilization phase (2005–2019), and a rapid growth phase from 2020 onward. The increasing engagement of authors from 88 countries further underscores the journal’s growing global appeal.

**Fig. 3:**
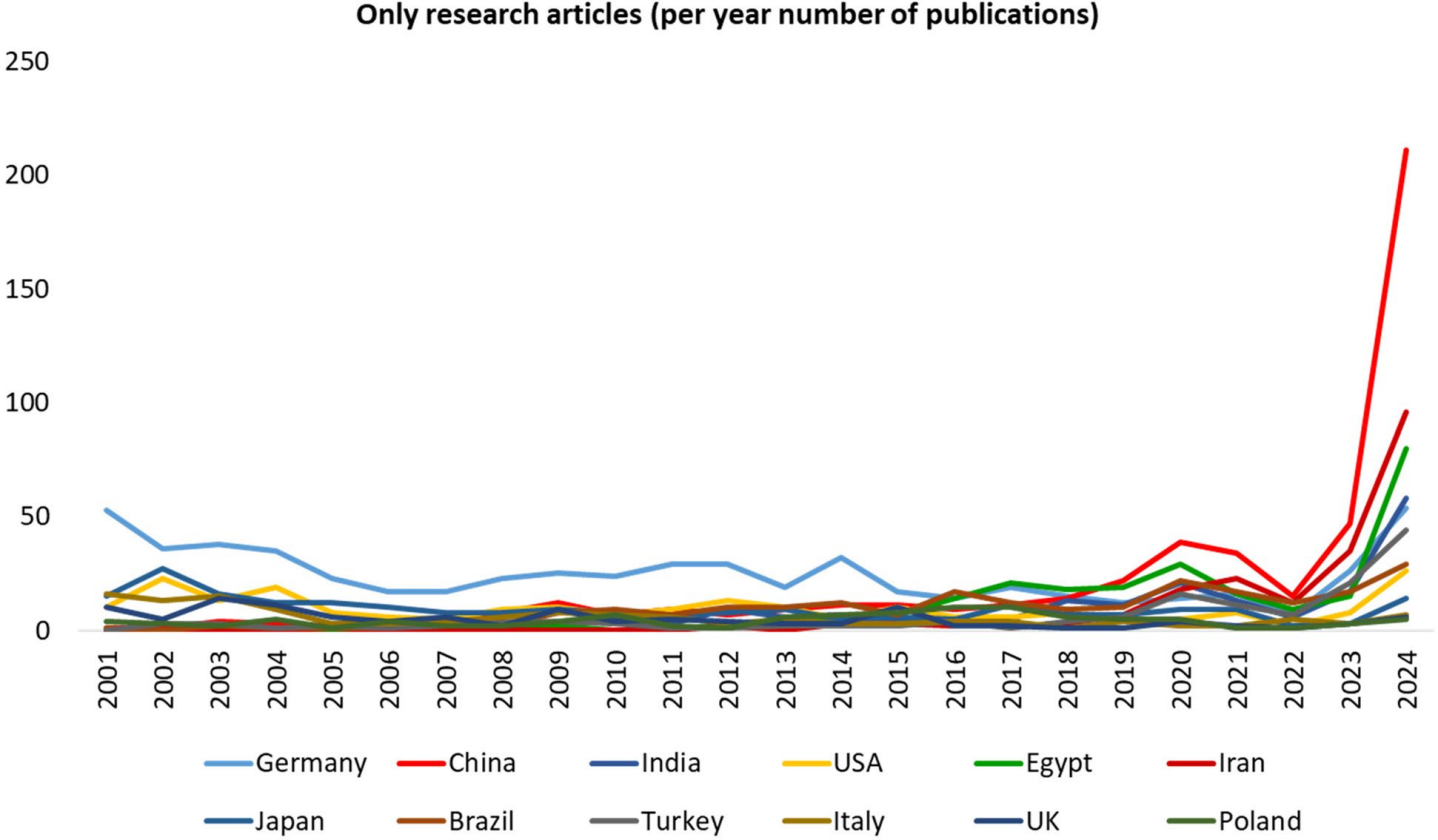
Annual number of original articles from countries with at least 100 publications inNaunyn–Schmiedeberg’s Archives of Pharmacology (NSAP), 2001–2024

The annual number of research article publications is presented in Supplementary Table 3. A list of countries with at least 100 research article publications is shown in Fig. 3. Overall, 85 countries contributed to research articles in NSAP. Detailed data on the number of publications and total citations for each country is provided in Supplementary Table 4.

#### Countries with 11–55 research articles (regression, ***y*** = 25.965***x***–175.76; ***R***.^2^ = 0.6575)

**Fig. 4:**
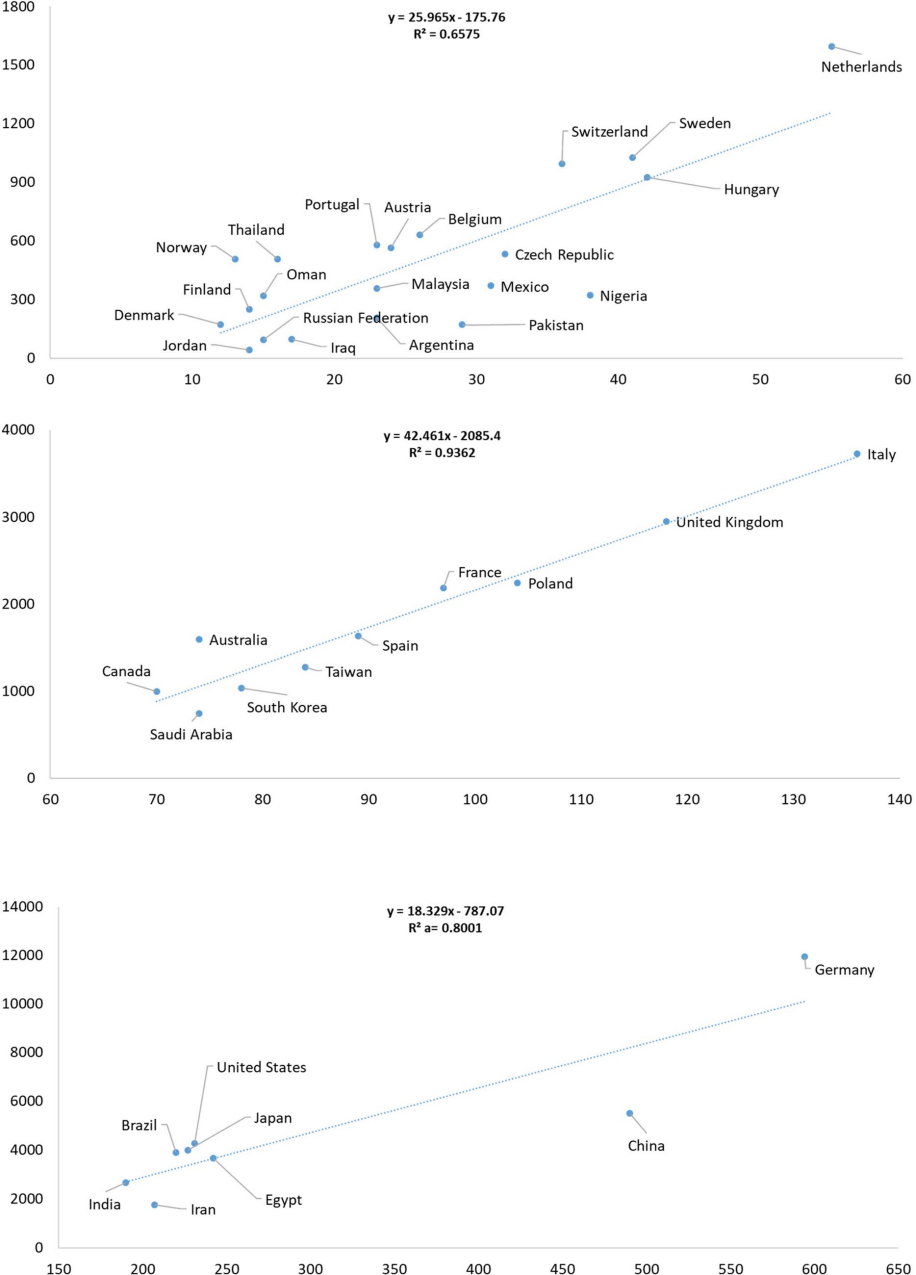
Linear regression analysis of countries, with the number of publications on the** x**-axis and total citations on the *y*-axis. The data includes only research articles published in *Naunyn–Schmiedeberg’s Archives of Pharmacology* (NSAP) from 2001 to 2024

Among the 21 countries that published between 11 and 55 research articles, citation performance varied considerably. This is illustrated in Fig. 4. Regression data for countries with fewer than 10 publications is presented in Supplementary Fig. 1.

Eleven countries stood above the regression line, suggesting a stronger-than-expected citation impact given their moderate publication counts. These included Norway (13 documents, 508 citations); Thailand (16, 506); Oman (15, 320); Finland (14, 249); Portugal (23, 578); Austria (24, 564); Belgium (26, 632); Switzerland (36, 995); Sweden (41, 1027); Netherlands (55, 1596); and Denmark (12, 173). Their above-average influence likely reflects a combination of strong institutional collaboration, high-impact research topics, and global visibility despite moderate output.

Conversely, six countries fell below the regression line, indicating a lower-than-expected citation return relative to their output. These were Jordan (14 documents, 43 citations); Russian Federation (15, 95); Argentina (23, 207); Malaysia (23, 358); Pakistan (29, 172); and Nigeria (38, 322). These results suggest potential barriers to citation reach, which may be due to limited international collaboration, less visibility in top-tier journals, or thematic focus areas with.

#### Countries with 70–136 research articles (regression, *y* = 42.461*x*–2085.4; R^2^ = 0.9362)

In the group of 11 countries producing between 70 and 136 research articles, the regression analysis revealed clear performance contrasts. Countries that outperformed the regression line included Canada (70 documents, 996 citations); Australia (74, 1594); France (97, 2186); the UK (118, 2947); and Italy (136, 3727)—all showcasing high citation yields relative to their substantial outputs. These countries benefitted from well-established pharmacological research systems, broad international collaboration, and consistent publication in influential outlets.

In contrast, three countries were positioned below the regression line, suggesting underperformance in terms of total number of citations: Saudi Arabia (74 documents, 743 citations); South Korea (78, 1037); and Taiwan (84, 1277). While their output was comparable to countries above the line, the lower citation counts reflect reduced relative impact, potentially due to differences in research topic visibility, journal selection, or global collaboration intensity.

#### Countries with 190–594 research articles (regression, ***y*** = 18.329*x*–787.07; R^2^ = 0.8001)

Among the eight countries with the highest research article output (190 to 594 papers), the regression model revealed that Brazil (220 documents, 3895 citations); Japan (227, 4001); the United States (231, 4278); and Germany (594, 11,952) performed above the regression line, signifying substantial citation influence relative to their large publication volumes. These countries demonstrate robust research ecosystems with global visibility and impactful outputs.

India (190 documents, 2659 citations) aligned closely with the regression line, indicating that its citation performance was proportional to its volume of publications. In contrast, Iran (207 documents, 1752 citations) and China (490, 5514) were below the regression line, revealing lower-than-expected citation outcomes. Despite their high productivity, both countries exhibited weaker citation returns, which may reflect challenges in international engagement, thematic targeting, or differences in journal impact levels.

### The total reviews in NSAP

Between 2001 and 2024, NSAP published a total of 505 review articles. In the early years, the number of reviews remained low, with only 1 review published in both 2001 and 2003, and slightly higher counts such as 5 reviews in 2002 and 10 reviews in 2004. A gradual increase was observed during the mid-2000s, with 12 reviews in 2005, 20 reviews in 2006, and 13 reviews each in 2007 and 2008. However, after a brief dip in 2009 and 2010, review publication became more consistent, reaching 18 reviews in 2011 and maintaining moderate numbers through the 2010s. A significant rise occurred after 2020, with 21 reviews published in 2021, 29 in 2022, and a dramatic surge to 62 reviews in 2023. In 2024, the journal published an extraordinary 204 review articles.

**Fig. 5:**
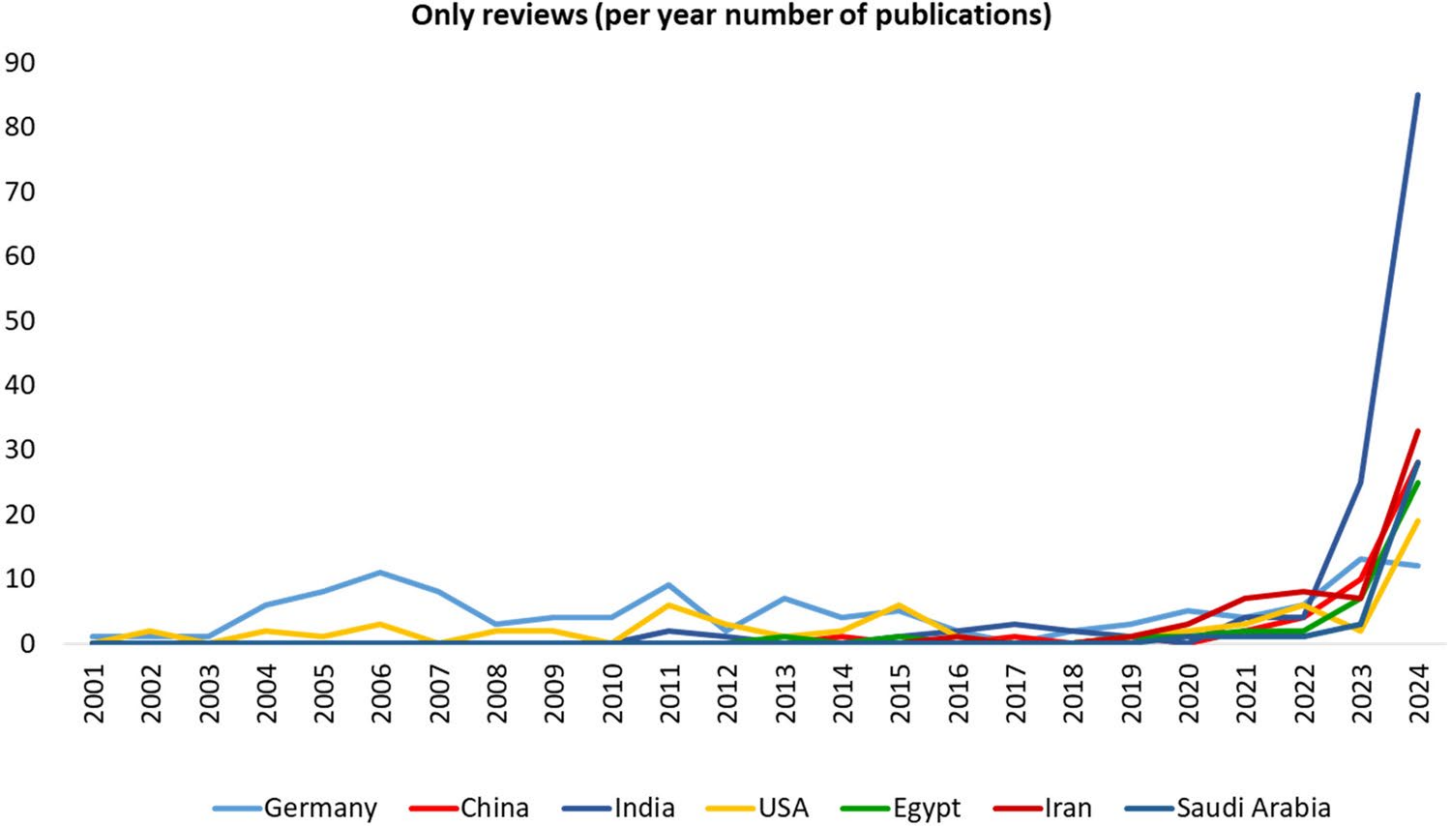
Annual number of review articles from countries with at least 100 publications in *Naunyn–Schmiedeberg’s Archives of Pharmacology* (NSAP), 2001–2024

**Fig. 6:**
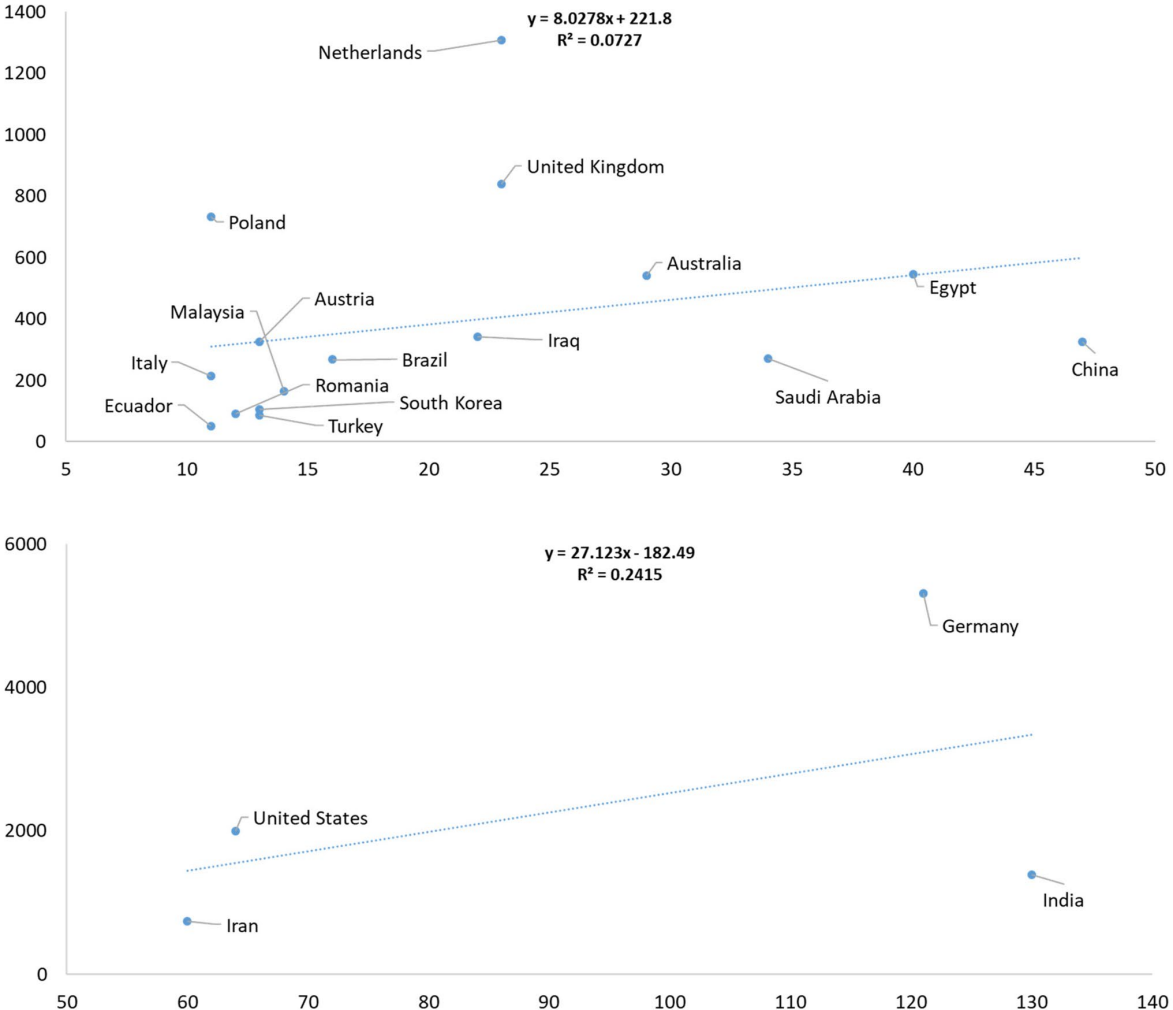
Linear regression analysis of countries, with the number of publications on the x-axis and total citations on the y-axis. The data includes only reviews published in *Naunyn–Schmiedeberg’s Archives of Pharmacology* (NSAP) from 2001 to 2024

The annual number of review articles is presented in Supplementary Table 5. Countries with at least 100 review publications with dynamics are shown in Fig. 5. In total, 75 countries contributed to review articles. Data for countries with more than 10 review publications is illustrated in Fig. 6, while regression analysis for countries with fewer than 10 publications is presented in Supplementary Fig. 3. Detailed information on the number of publications and total citations by country is provided in Supplementary Table 6.

#### Countries with 11–47 publications

The following regression-based observations pertain to the 505 review articles published in NSAP. Among these, 16 (*n* = 16) countries contributed between 11 and 47 publications, with a regression line showing a very low explanatory power (*R*^2^ = 0.0727). This weak correlation indicates that the number of reviews published by a country had little predictive value for citation performance within this group. Nonetheless, a subset of countries performed above the regression line, suggesting greater-than-expected citation impact. These included Poland (11 documents, 732 citations); Austria (13 documents, 326 citations); Netherlands (23 documents, 1308 citations); the UK (23 documents, 840 citations); Australia (29 documents, 540 citations); and Egypt (40 documents, 546 citations). Their performance points to efficient dissemination, strong topic relevance, or enhanced research visibility.

In contrast, several countries fell below the regression line, signaling lower-than-expected citation returns. These were Ecuador (11 documents, 51 citations); Brazil (16 documents, 269 citations); Malaysia (14 documents, 165 citations); South Korea (13 documents, 106 citations); Iraq (22 documents, 343 citations); Saudi Arabia (34 documents, 271 citations); and China (47 documents, 326 citations). Their underperformance may reflect challenges in visibility, limited international collaboration, or choice of publication topics.

#### Countries with 60–130 publications

For countries with higher publication volume, a second group comprising four (*n* = 4) countries—each contributing between 60 and 130 review articles—was also analyzed. Here, the regression model demonstrated a weak association (*R*^2^ = 0.241) between publication count and citation output, but some notable differences emerged. Above the regression line, United States (64 documents, 2000 citations) and Germany (121 documents, 5314 citations) demonstrated strong citation performance, highlighting their global research prominence and high-impact review output. Meanwhile, Iran (60 documents, 739 citations) and India (130 documents, 1388 citations) fell below the regression line, indicating lower citations despite substantial output. This disparity suggests structural or qualitative differences in the reach and influence of review articles across nations.

Overall, these findings underline how citation performance in NSAP reviews is influenced not merely by output volume, but also by factors such as topic salience, institutional prestige, and global collaboration networks.

### Discussion

One possible explanation for the relatively low citation rates of papers from certain countries is a growing lack of confidence among readers, particularly due to rising awareness of fraudulent submissions linked to paper mills. These entities produce and disseminate fabricated research, undermining the credibility of scholarly literature and distorting academic metrics.

NSAP has also faced challenges from such unethical practices. In 2020 and 2021, the journal retracted 11 articles identified as originating from paper mills (Seifert 2021). With approximately 1000 submissions received annually in 2019 and 2020, it is estimated that up to 5% of these may have been associated with such sources. The consequences are far-reaching, as paper mills compromise the integrity of the peer-review process, misrepresent authorship, and potentially influence citation patterns.

A more extensive analysis by Wittau et al. (2024) reviewed 2056 withdrawn or rejected submissions to NSAP. Of these, 952 were later published in other journals, and in some cases, the list of authors had changed substantially—by over two-thirds in six cases, and entirely in four. This manipulation highlights how paper mills exploit the lack of transparency and coordination between journals, allowing for multiple submissions and author substitutions.

When checked on 11 June, 2025, NSAP has retracted 42 papers with a notable concentration from a few countries: China (15), Egypt (11), Pakistan (6), India (4), and Iran (3). Between 2000 and 2023, the overall retraction rate in NSAP was 1.16% which is very low compared to other journals (van Diest et al. 2025). Although these retraction instances do not represent the entirety of research output from these nations, such patterns may contribute to increased skepticism from reviewers and readers alike. The list of 42 retracted papers, the universities with at least two retractions, and the country-wise distribution of retracted papers are provided in Supplementary Tables 7–9, respectively. This erosion of trust may, in turn, affect the visibility and citation performance of research from these regions.

To address this issue, countries must take firm and transparent actions to discourage or block the operations of paper mills, which produce fraudulent or low-quality manuscripts and sell authorship slots. Notably, China’s Supreme People’s Court has called for a nationwide crackdown on such activities in an effort to protect the integrity of scientific research (Mallapaty, 2025).

NSAP is not the only journal reflecting relatively low citation impact from low-income or low- and middle-income countries (LMICs). Our group has recently identified similar disparities in other prominent and well-established journals. For instance, notable imbalances in publications and citation metrics from LMICs have been reported in *Nitric Oxide–Biology and Chemistry* [Hassan et al., 2022], *Pharmacological Research* (Hassan et al., 2021), *Food Chemistry* (Kamdem et al., 2019), and *Chemico-Biological Interactions* (Hassan et al. 2020). These findings repeatedly highlight systemic obstacles that limit the visibility and academic contribution of researchers from LMIC regions across several prestigious journals.

This underrepresentation is not confined to pharmacology or chemistry-related fields. Previous investigations by Sumathipala et al. (2004) and Woods et al. (2023) documented similar trends in five leading medical journals—*British Medical Journal*, *The Lancet*, *New England Journal of Medicine (NEJM)*, *Annals of Internal Medicine*, and *Journal of the American Medical Association (JAMA)*. These studies pointed to persistent ethical and structural barriers that restrict equitable participation of LMIC researchers in high-impact scientific publishing (Sumathipala et al. 2004; Woods et al., 2023).

Disparities have also been identified across various targeted research areas. For example, Servadei et al. (2019) noted the limited representation of LMIC scholars in neurosurgical research. In nurse-led cancer research, Molassiotis et al. (2024) reported minimal LMIC involvement despite the field’s growth. A similar gap was found in perinatal depression studies, as shown by Dol et al. (2024). In orthopaedic surgery, Sabharwal et al. (2024) conducted a comparative study revealing a stark publication gap from lower-income regions. Mukku et al. (2025) demonstrated that the literature on behavioral and psychological symptoms of dementia (BPSD) is largely driven by high-income countries, with little contribution from LMICs. Additional disparities were evident in research on aneurysmal subarachnoid hemorrhage (da Costa Borsatto et al., 2023) and gynecologic oncology (Levin et al. 2023), both of which revealed low levels of LMIC involvement and reduced citation impact for those contributions.

Similarly, the disparities are also cited by the *Nature Index*. For instance, they presented a detailed analysis (in 2023) of research productivity across East and Southeast Asia, as well as Oceania, including Australia and New Zealand (Woolston 2023). Notably, the report underscored China’s surpassing of the United States in the volume of high-impact scientific publications—particularly within the physical and chemical sciences.

Continuing this trend, India has also gained ground. As highlighted in the *Nature Index 2024 Research Leaders* (formerly known as the Annual Tables), India has advanced to 9th place globally, overtaking both Australia and Switzerland (nature.com 2025). In the field of natural sciences, it now ranks 8th overall, demonstrating its rising influence in global research output.

More recent data from *Nature Index 2025* suggest a further shift in scientific dominance (nature.com 2025). The United States has experienced a substantial decline in its adjusted share, particularly in chemistry (down 11.6%) and the physical sciences (down 10.6%). While it still leads in the health sciences, its decrease there was more modest (2.7%). The US continues to hold the top spot in biological sciences, yet its adjusted share in that field fell by 5.4%, whereas China’s share rose sharply—by over 20%. This expansion has also affected other major Western research systems: Canada, France, and the UK each experienced declines in adjusted share.

Taken together, these studies show that the underrepresentation of LMICs is not limited to isolated journals or subfields. It reflects a widespread pattern affecting numerous disciplines across medicine and health research. Researchers in LMICs continue to face structural disadvantages that slow their progress relative to high-income countries (HICs). These disadvantages are interrelated and include institutional weaknesses, limited funding, language barriers, and geopolitical challenges.

One of the most significant obstacles is the low level of financial investment in research and development. Many LMICs allocate a very small portion of their gross domestic product (GDP) to this sector. For example, several African Union nations spend less than 0.5% of GDP on research, falling short of global benchmarks (Simpkin et al. 2019). This chronic underfunding undermines research infrastructure, limits access to digital resources, and restricts availability of tools such as statistical software and reliable internet (Anane-Binfoh et al. 2024).

Language barriers further intensify these difficulties. The dominance of English in academic publishing places non-native speakers at a disadvantage when applying for grants, preparing manuscripts, and engaging in scholarly communication (Dakhil et al. 2024). High publication fees and the lack of institutional editorial support further reduce the visibility of LMIC research.

Additional challenges affect researchers in specific regions such as the Arab world, where gaps in mentorship, collaborative culture, and institutional encouragement diminish research productivity and reduce the likelihood of publication in competitive journals (Elgamri et al. 2023).

These interconnected challenges—limited financial resources, language-related disadvantages, infrastructural deficiencies, and weak research networks—are key contributors to the slower pace of research development in LMICs compared to their high-income counterparts.

Alemayehu et al. (2018) made a valuable contribution by analyzing the key barriers that hinder clinical trial activity in LMICs. Their review identified core problem areas, each supported by relevant references and practical examples.

1. The first challenge, related to financial and human capacity, included chronic underfunding, shortages of skilled personnel, and low motivation among potential researchers. These factors limit the ability to plan and conduct high-quality clinical trials.
2. Second, the authors noted difficulties in ethical and regulatory systems, such as long delays in approvals, involvement of underqualified regulatory staff, and overly complex ethical review processes. These challenges discourage investigator-led research and create inefficiencies.
3. Third, the lack of a conducive research environment was emphasized. This included weak infrastructure, limited access to essential materials, and the absence of a stimulating academic setting.
4. Fourth, operational issues were identified, including administrative inefficiencies and problems with patient recruitment.
5. Fifth, the authors pointed to time pressures and competing responsibilities as key obstacles that prevent clinicians and academics from prioritizing research.
6. Altogether, these findings reveal the depth and variety of structural limitations that must be addressed to improve clinical research in LMICs.

In a related effort, Sharma et al. (2024) provided a well-structured set of recommendations for strengthening research capacity in LMICs. Their proposals targeted government, institutional, academic, and international domains and highlighted the importance of a long-term, coordinated response.

1. They stressed that governments and academic institutions should define clear research priorities and allocate dedicated funding to support them. A fixed percentage of national and institutional budgets should be committed to research.
2. The authors also advocated for the integration of research training from an early age—beginning in school and continuing through higher education—to help create a lasting culture of inquiry. Public awareness campaigns were recommended to build societal recognition of research value.
3. Academic and clinical institutions were encouraged to formally acknowledge the role of research in healthcare improvement. Recommendations included establishing full- and part-time research positions, assigning protected research hours for academic staff, and adopting merit-based evaluation systems that reward quality over quantity.
4. To build a skilled research workforce, Sharma et al. proposed scholarships for postgraduate and postdoctoral training and investment in high-quality local and international mentorship programs. They also supported the development of interdisciplinary collaborations to enrich research depth and broaden its applications.
5. On the publishing front, they urged journals to offer editorial support to LMIC authors and to include editors and reviewers who understand local priorities. Addressing language barriers was another key recommendation, with suggestions for assistance from native English speakers during manuscript preparation.

In summary, Sharma et al. (2024) outlined a comprehensive framework that not only identifies persistent barriers in LMIC research but also offers specific, actionable steps to enhance visibility, capacity, and impact in global science.

The sharp rise in total publications from 2020 onward—peaking at 909 publications and 665 original articles in 2024—indicates a significant transformation in NSAP’s international perception as an important journal. The dramatic increase in review articles (204 in 2024 alone) was initially welcome by the Editor-in-Chief, but it remains to be determined to which extent the recent surge of review papers in NSAP is due to the non-declared use of large language models by authors. As a consequence of this development, NSAP has recently implemented an editorial policy, not allowing the use of artificial intelligence technology at all (Seifert et al. 2025).

Original research articles (3386; 81.5% of total output) remain the core of NSAP’s scholarly contribution. In view of the potential problems associated with artificial intelligence-generated reviews, the focus of the journal will stay on research articles although in average, research papers receive fewer citations than reviews and are "unfavorable" in terms of the journal impact factor. But the focus on integrity is at a premium. 

NSAP’s global footprint is broad, with authors from 95 countries overall and 85 contributing original research. Despite this internationalization, citation distributions remain unequal. High-income countries (HICs) such as Germany, the United States, Japan, and Western European nations consistently outperformed others in terms of total citations. In contrast, upper-middle-income (UMICs) and lower-middle-income countries (LMICs)—including China, India, Egypt, Iran, and Brazil—exhibited weaker citation returns despite high publication volumes, indicating persistent barriers in visibility, collaboration, or perceived research quality.

A regression-based analysis categorizing countries into low-, mid-, and high-volume tiers revealed critical insights into national performance:

1. Positive outliers such as Sweden, Switzerland, and Belgium showed strong citation performance relative to modest publication output, demonstrating research efficiency.
2. Underperformers like Malaysia, Pakistan, and Jordan repeatedly fell below expected impact thresholds, raising concerns about global integration and citation outreach.
3. The high *R*^2^ values (e.g., up to 0.93 for mid-tier research articles) affirm the model’s reliability for benchmarking country-level research engagement and guiding editorial strategy.

While NSAP has succeeded in attracting a diverse international author base, this inclusivity has not yet translated into equitable citation impact. The ongoing disparity—where increased contributions from LMICs and UMICs do not correspond to proportional citation gains—signals a need for structural interventions to enhance global research visibility, mentorship, and citation equity.

The journal’s rising publication volume and international contributions reflect its growing role in global pharmacological research dissemination. However, to sustain impact and avoid dilution, NSAP must enforce a careful balance between inclusivity and quality control. Strategic editorial planning is essential to ensure that increased participation enhances rather than undermines the journal’s influence.

NSAP’s publication and citation trends offer a valuable dataset for bibliometric and policy research. The integration of regression models,* R*^2^ values, and country-specific performance assessments provides a systematic approach for evaluating national research efficiency. These insights can inform journal editors, funding agencies, and academic institutions in developing targeted strategies to strengthen research quality and global integration.

### Conclusion

This comprehensive bibliometric analysis of NSAP from 2001 to 2024 reveals three critical trends: (1) a sharp increase in publication volume, particularly in the last 2 years; (2) considerable international participation; and (3) wide variation in total number of citations across countries and document types.

The journal published 4155 documents during this period, predominantly original research articles (3386), with notable increases in review articles, particularly in 2023–2024. After a period of relative stability (2001–2019), a dramatic surge in output occurred from 2020 onward, peaking with 909 total publications and 665 research articles in 2024.

Ninety-five countries contributed to NSAP’s total output, with Germany, China, India, the United States, and Egypt being the most productive. Regression-based country analyses across three tiers (low, mid, high productivity) revealed substantial disparities in citation performance. High-income countries such as Germany, the US, and Japan consistently exceeded citation expectations, while several LMICs and UMICs—despite substantial publication volumes—demonstrated lower citations, highlighting gaps in global research visibility, impact, or collaboration.

Original research articles and reviews followed similar trends. Highly cited countries again included Germany, the UK, and the Netherlands, whereas China, Iran, and several LMICs produced large volumes with comparatively modest citation returns. In contrast, countries like Switzerland, Sweden, and Belgium often outperformed expectations with smaller outputs but high citation impact, demonstrating effective research dissemination.

In summary, NSAP has significantly expanded its publishing capacity in recent years and continues to attract a globally diverse set of authors. However, disparities in citation impact underscore the importance of not just increasing volume but also enhancing quality, visibility, and international collaboration—particularly for emerging research nations.

### Implications for NSAP policies

NSAP is committed to publishing the best science, regardless of geographic origin. This editorial policy should encourage authors from all countries to submit their work to our journal. Authors can rest assured that editors have no conscious biases in favor or against authors from a given country. An argument in favor of this notion is that papers received from, e.g., China, are processed through peer review in a similar time frame as papers from other countries including Germany. Evidently, a journal is not in the position to affect science policies in different countries. But if authors rigorously implement the suggestions by referees and editors of NSAP and do not consider them as a kind of penalty or undue extra burden, the science presented in the paper will get better and in the long run, these papers will get cited more frequently. Authors coming from countries in which paper mills are active, should do whatever they can to abide to professional conduct in research and join the journal in its mission for high scientific standards. Submitting “minimally publishable unit” papers to NSAP is strongly discouraged and will not lead to high citations of papers from a given country. Lastly, trying to rush through a paper revision process “quick and dirty” is not a successful strategy to increase the recognition, reputation and citation of science from a country. Thus, authors form LMIC und UMCI have it in their hands to do their share to increase their citation rates. NSAP will support these authors by implementing high scientific standards in the peer review process.

### Limitations

This study has several important limitations that should be acknowledged: Our analysis is based solely on publications from *Naunyn–Schmiedeberg’s Archives of Pharmacology* (NSAP), which does not comprehensively reflect the overall scientific output or productivity trends of all mentioned countries. The choice of journal submission is influenced by multiple factors including editorial policy, author preference, institutional incentives, and visibility goals, all of which may introduce selection bias. Therefore, extrapolating findings from one journal to broader national productivity patterns must be interpreted with caution.

As citation accumulation is inherently time-sensitive, papers published earlier have had more opportunity to garner citations. Given the evolving publication patterns over the past two decades—with some countries increasing their output only recently—there is a risk of overestimating the citation performance of countries that were more active earlier in the study period. We did not apply statistical adjustments (e.g., time normalization or fixed-year citation windows) to control for this bias, which may affect the comparability of citation metrics across countries.

Similarly, the study correlated total citations with total publication counts, which may not fully capture disparities in citation impact. Citation distributions are typically skewed and non-normal, making parametric approaches such as linear regression potentially inappropriate. We acknowledge that calculating median citations and interquartile ranges (IQRs) per paper, as well as employing non-parametric statistical techniques, would provide a more accurate and statistically robust comparison across countries.

We also recognize that variations in citation frequency across countries can stem from multiple sources not fully explored in our analysis. These include cultural or regional citation practices (“citation bias”) as well as differences in research quality, visibility, collaboration networks, and journal impact. These complex and interrelated factors require deeper qualitative and quantitative investigation. Although discussed in prior literature, these issues were not extensively cited or integrated into our discussion.

NSAP publishes across a range of pharmacological subfields, and countries may differ in their thematic focus. Citation rates can vary substantially across disciplines, and our study did not normalize for topic or field-specific citation behavior. This could substantially influence the interpretation of country-level citation averages. In summary, while the findings offer a snapshot of geographical disparities in publication and citation dynamics within NSAP, they should be interpreted as exploratory. Broader generalizations about national research productivity or impact would require a multi-journal, multi-field, and time-adjusted framework.

## Supplementary Information

Below is the link to the electronic supplementary material.

Supplementary file 1 (DOCX 49.1 KB)

Supplementary file 2 (DOCX 216 KB)

## Data Availability

All source data of this research project are available from the authors upon reasonable request.

## Abbreviations

NSAP: Naunyn-Schmiedeberg’s Archives of Pharmacology
HICs: High-Income Countries
UMICs: Upper-middle-income countries
LMICs: Lower-middle-income countries
GSP: Good scientific practice
NIH: National Institutes of Health
RCR: Responsible conduct of research
NHMRC: National Health and Medical Research Council
ARC: Australian Research Council
NEJM: New England Journal of Medicine
JAMA: Journal of the American Medical Association

## References

[CR1] Abdelwahab SI, Farasani A, Moshi JM et al (2025a) Authorship analysis of publications in *Naunyn–Schmiedeberg’s Archives of Pharmacology* (1969–2024). Naunyn-Schmiedeberg’s Arch Pharmacol. 10.1007/s00210-025-04253-1 10.1007/s00210-025-04253-1 40387930

[CR2] Abdelwahab SI, Farasani A, Moshi JM et al (2025b) Exploring the presence of DFG-funded publications in Naunyn-Schmiedeberg’s Archives of Pharmacology. Naunyn-Schmiedeberg’s Arch Pharmacol. 10.1007/s00210-025-04254-0 10.1007/s00210-025-04254-0 40343451

[CR3] Abdelwahab SI, Farasani A, Moshi, JM et al (2025c) Germany in pharmacology publishing: 192 articles with 1367 citations in Naunyn–Schmiedeberg’s Archives vs. 16,775 articles with 274,225 citations elsewhere. Naunyn-Schmiedeberg’s Arch Pharmacol. 10.1007/s00210-025-04224-6 10.1007/s00210-025-04224-6 40310531

[CR4] Alemayehu C, Mitchell G, Nikles J (2018) Barriers for conducting clinical trials in developing countries—a systematic review. Int J Equity Health 17(1):37. 10.1186/s12939-018-0748-6 29566721 10.1186/s12939-018-0748-6 PMC5863824

[CR5] Anane-Binfoh NA, Flaherty KE, Zakariah AN, Nelson EJ, Becker TK, Afaa TJ (2024) Barriers to decolonizing global health: identification of research challenges facing investigators residing in low- and middle-income countries. Global Health: Science and Practice 12(1):e2300269. 10.9745/GHSP-D-23-00269 38242635 10.9745/GHSP-D-23-00269 PMC10906550

[CR6] Basol ME, Seifert R (2024) Bibliometric analysis of Naunyn–Schmiedeberg’s Archives of Pharmacology (1947–1974). Naunyn–Schmiedeberg's Arch Pharmacol 397(9):7141–7168 10.1007/s00210-024-03078-8 PMC11422447 38652278

[CR7] Bünemann S, Seifert R (2024) Bibliometric comparison of Nobel Prize laureates in physiology or medicine and chemistry. Naunyn-Schmiedebergs Arch Pharmacol 397:7169–7185. 10.1007/s00210-024-03081-z 38652280 10.1007/s00210-024-03081-z PMC11422443

[CR8] da Costa Borsatto GJ, Bertelli Ramos M, Mota Telles JP, Nunes Rabelo N, Jacobsen Teixeira M, Gadelha FE (2023Jul 5) Research trends within aneurysmal subarachnoid hemorrhage from 2017 to 2021: a bibliometric study. Neurosurg Rev 46(1):165. 10.1007/s10143-023-02056-7 37405510 10.1007/s10143-023-02056-7

[CR9] Dakhil ZA, Cader FA, Banerjee A (2024) Challenges in clinical research in low and middle income countries: early career cardiologists’ perspective. Glob Heart 19(1):13. 10.5334/gh.1293 38273996 10.5334/gh.1293 PMC10809862

[CR10] Dats LB, von Haugwitz F, Seifert R (2023) Bibliometric development of Naunyn–Schmiedeberg’s archives of pharmacology. Naunyn Schmiedebergs Arch Pharmacol 396(1):43–61 36280660 10.1007/s00210-022-02307-2 PMC9592544

[CR11] Dol J, Dennis CL, Campbell-Yeo M, Leahy-Warren P (2024Mar) Bibliometric analysis of published articles on perinatal depression from 1920 to 2020. Birth 51(1):28–38. 10.1111/birt.12779 37795646 10.1111/birt.12779

[CR12] Elgamri A, Mohammed Z, El-Rhazi K, Shahrouri M, Ahram M, Al-Abbas A-M, Silverman H (2023) Challenges facing Arab researchers in conducting and publishing scientific research: a qualitative interview study. Research Ethics 20(2):331–362. 10.1177/17470161231214636

[CR13] Fox LC, Seifert R (2024) Arbitrariness of bibliometric parameters: a case study on leading scientists in the German Society for Experimental and Clinical Pharmacology and Toxicology (DGPT). Naunyn-Schmiedebergs Arch Pharmacol 397:8925–8942. 10.1007/s00210-024-03195-4 38864907 10.1007/s00210-024-03195-4 PMC11639183

[CR14] Halling T, Mambrey V, Steinert J et al (2025) The Gender Award Gap in German medical societies 2000–2023: the Fritz-Külz-Award as an example. Naunyn-Schmiedeberg’s Arch Pharmacol. 10.1007/s00210-025-03892-8 10.1007/s00210-025-03892-8 PMC12350518 40047859

[CR15] Hassan W, Kamdem JP, Teixeira da Rocha JB (2020) Research trends in chemico-biological interactions: the golden jubilee (1969–2019). Chem Biol Interact 327:109177. 10.1016/j.cbi.2020.109177 10.1016/j.cbi.2020.109177 32533983

[CR16] Hassan W, Zafar M, Duarte AE, Kamdem JP, Teixeira da Rocha JB (2021) Pharmacological research: a bibliometric analysis from 1989 to 2019. Pharmacol Res 169:105645. 10.1016/j.phrs.2021.105645 10.1016/j.phrs.2021.105645 33957268

[CR17] Hassan W, Zafar M, Duarte AE, Kamdem JP, Teixeira da Rocha JB (2022) The silver jubilee of the nitric oxide journal: from 1997 to 2021. Nitric Oxide 124:74–87. 10.1016/j.niox.2022.05.003 10.1016/j.niox.2022.05.003 35644419

[CR18] Hassan W, Abdelwahab SI, Farasani A et al (2025) From Germany to the world: analysis of 25,931 documents (from 1873 to 2025) published in Naunyn–Schmiedeberg’s archives of pharmacology. Naunyn-Schmiedebergs Arch Pharmacol. 10.1007/s00210-025-04199-4 40317317 10.1007/s00210-025-04199-4

[CR19] Hattori Y, Seifert R (2023) Reflections on the 150th anniversary of Naunyn–Schmiedeberg’s archives of pharmacology: past, challenges, and future. Naunyn Schmiedebergs Arch Pharmacol 396(1):1–3 36336742 10.1007/s00210-022-02321-4 PMC9788996

[CR20] https://www.nature.com/nature-index/news/nature-index-research-leaders-india-follows-china-footsteps. Accessed 10 Jul 2025

[CR21] https://www.nature.com/nature-index/research-leaders/2025/nature-index-2025-research-leaders-united-states-losing-ground-as-chinas-lead-expands-rapidly.html. Accessed 10 Jul 2025

[CR22] Steinert JM, Seifert R (2025) The Schmiedeberg Medal of the German Society for Experimental and Clinical Pharmacology and Toxicology: a biographical and bibliometric analysis of the 47 recipients from (1956) to 2024. Naunyn Schmiedebergs Arch Pharmacol. 10.1007/s00210-025-04260-2 10.1007/s00210-025-04260-2 PMC12678592 40481848

[CR23] Kamdem JP, Duarte AE, Lima KRR, Rocha JBT, Hassan W, Barros LM, Roeder T, Tsopmo A (2019Oct) research trends in food chemistry: a bibliometric review of its 40 years anniversary (1976–2016). Food Chem 1(294):448–457. 10.1016/j.foodchem.2019.05.021 10.1016/j.foodchem.2019.05.021 31126486

[CR24] Levin G, Pareja R, Harrison R, Ramirez PT, Meyer R (2023) Association of literature metrics in gynecologic oncology with country classification by income level. Int J Gynecol Cancer 33(6):957–963. 10.1136/ijgc-2023-004380 37001895 10.1136/ijgc-2023-004380

[CR25] Mallapaty S (2025Mar) China’s supreme court calls for crack down on paper mills. Nature 639(8054):285–286. 10.1038/d41586-025-00612-3 40038497 10.1038/d41586-025-00612-3

[CR26] Molassiotis A, Yorke J, McCarthy AL, Wengstrom Y, Gibson F, Abu-Odah H (2024) The evolution of worldwide nurse-led cancer research in the last 2 decades (2004–2022): a bibliometric mapping and visual analysis. Cancer Nursing 47(5):E308-E317. 10.1097/NCC.0000000000001260 10.1097/NCC.0000000000001260 37552219

[CR27] Mukku SSR, Darshanam V, Dahale AB (2025) A bibliometric analysis of 100 most-cited articles on behavioural and psychological symptoms of dementia (BPSD). Asian J Psychiatr 107:104462. 10.1016/j.ajp.2025.104462 40185049 10.1016/j.ajp.2025.104462

[CR28] Sabel BA, Seifert R (2021) How criminal science publishing gangs damage the genesis of knowledge and technology—a call to action to restore trust. Naunyn Schmiedebergs Arch Pharmacol 394(11):2147–2151. 10.1007/s00210-021-02158-3 34554267 10.1007/s00210-021-02158-3 PMC8514363

[CR29] Sabharwal S, Leung A, Rodarte P, Singh G, Bwemelo JJ, Taylor AS, Tan J, Trott R (2024) Peer-reviewed publications in orthopaedic surgery from lower income countries: a comparative analysis. SICOT J 10:6. 10.1051/sicotj/2023039 38305681 10.1051/sicotj/2023039 PMC10836199

[CR30] Seifert R (2021) How Naunyn-schmiedeberg’s archives of pharmacology deals with fraudulent papers from paper mills. Naunyn-Schmiedeberg’s Arch Pharmacol 394:431–436. 10.1007/s00210-021-02056-8 33547901 10.1007/s00210-021-02056-8 PMC7865115

[CR31] Seifert R, Hartman E, Wang K et al (2025) Authors must follow the editorial guidelines on the use of large language models in review papers. Naunyn-Schmiedeberg’s Arch Pharmacol. 10.1007/s00210-025-04102-1 10.1007/s00210-025-04102-1 PMC12263706 40156609

[CR32] Servadei F, Tropeano MP, Spaggiari R, Cannizzaro D, Al Fauzi A, Bajamal AH, Khan T, Kolias AG, Hutchinson PJ (2019) Footprint of reports from low- and low- to middle-income countries in the neurosurgical data: a study from 2015 to 2017. World Neurosurgery 130(1):e822–e830. 10.1016/j.wneu.2019.06.230 31295603 10.1016/j.wneu.2019.06.230

[CR33] Sharma S, Verhagen A, Elkins M, Brismée JM, Fulk GD, Taradaj J, Steen L, Jette A, Moore A, Stewart A, Hoogenboom BJ, Söderlund A, Harms M, Zambelli Pinto R (2024) Research from low-income and middle-income countries will benefit global health and the physiotherapy profession, but it requires support. J Neurol Phys Ther 48(1):1–5. 10.1097/NPT.0000000000000461 37772740 10.1097/NPT.0000000000000461 PMC10720871

[CR34] Simpkin V, Namubiru-Mwaura E, Clarke L, Mossialos E (2019) Investing in health R&D: where we are, what limits us, and how to make progress in Africa. BMJ Glob Health 4(2):e001047. 10.1136/bmjgh-2018-001047 30899571 10.1136/bmjgh-2018-001047 PMC6407556

[CR35] Sumathipala A, Siribaddana S, Patel V (2004) Under-representation of developing countries in the research literature: ethical issues arising from a survey of five leading medical journals. BMC Med Ethics 5(1):1–6. 10.1186/1472-6939-5-5 10.1186/1472-6939-5-5 PMC524359 15461820

[CR36] van Diest RA, Seifert R, van der Heyden MAG (2025) An extra pair of eyes: adopting innovative approaches to detect integrity issues in Naunyn-Schmiedeberg’s archives of pharmacology. Naunyn Schmiedebergs Arch Pharmacol 398(1):1–8. 10.1007/s00210-024-03697-1 39751822 10.1007/s00210-024-03697-1 PMC11787271

[CR37] Wittau J, Celik S, Kacprowski T et al (2024) Fake paper identification in the pool of withdrawn and rejected manuscripts submitted to *Naunyn–Schmiedeberg’s archives of pharmacology*. Naunyn-Schmiedeberg’s Arch Pharmacol 397:2171–2181. 10.1007/s00210-023-02741-w 37796310 10.1007/s00210-023-02741-w PMC10933159

[CR38] Woods WA, Watson M, Ranaweera S, Tajuria G, Sumathipala A (2023Jan) Under-representation of low and middle income countries (LMIC) in the research literature: ethical issues arising from a survey of five leading medical journals: have the trends changed? Glob Public Health 18(1):2229890. 10.1080/17441692.2023.2229890 37401751 10.1080/17441692.2023.2229890

[CR39] Woolston C (2023Jun 15) Nature Index Annual Tables 2023: China tops natural-science table. Nature. 10.1038/d41586-023-01868-3 37322250 10.1038/d41586-023-01868-3

[CR40] Zehetbauer R, von Haugwitz F, Seifert R (2022) Gender-specific analysis of the authors and the editorial board of Naunyn–Schmiedeberg’s archives of pharmacology from 2000 to 2020. Naunyn Schmiedebergs Arch Pharmacol 395(1):39–50 34622307 10.1007/s00210-021-02166-3 PMC8497184

[CR41] Zöllner H, Seifert R (2024) How do German pharmacologists publish in the non-peer-reviewed science magazine Biospektrum? Naunyn-Schmiedebergs Arch Pharmacol 397:1889–1900. 10.1007/s00210-023-02740-x 37776381 10.1007/s00210-023-02740-x PMC10858829

