# Supplementary material for: Country-specific citation disparities in Naunyn–Schmiedeberg’s Archives of Pharmacology from 2001 to 2024
Source: Naunyn Schmiedebergs Arch Pharmacol. 2025 Aug 14;399(2):2113–28. doi: 10.1007/s00210-025-04499-9 (PMC12901205; doi:10.1007/s00210-025-04499-9)
Supplement: Supplementary file 1 — (DOCX 49.1 KB) [file 210_2025_4499_MOESM1_ESM.docx]

**Legends**

1. **Supplementary Table 1:** Annual number of publications (all document types) in *Naunyn-Schmiedeberg’s Archives of Pharmacology*, 2001–2024.
2. **Supplementary Table 2:** List of 95 countries with publication and citation counts (all document types) in *Naunyn-Schmiedeberg’s Archives of Pharmacology*, 2001–2024.
3. **Supplementary Table 3:** Annual number of original research articles published in *Naunyn-Schmiedeberg’s Archives of Pharmacology*, 2001–2024.
4. **Supplementary Table 4:** List of 85 countries with publication and citation counts for original research articles in *Naunyn-Schmiedeberg’s Archives of Pharmacology*, 2001–2024.
5. **Supplementary Table 5:** Annual number of review articles published in *Naunyn-Schmiedeberg’s Archives of Pharmacology*, 2001–2024.
6. **Supplementary Table 6:** List of 75 countries with publication and citation counts for review articles in *Naunyn-Schmiedeberg’s Archives of Pharmacology*, 2001–2024.
7. **Supplementary Table 7:** List of 42 retracted papers published in *Naunyn-Schmiedeberg’s Archives of Pharmacology*.
8. **Supplementary Table 8:** Institutions with at least two publications among the 42 retracted papers in *Naunyn-Schmiedeberg’s Archives of Pharmacology*.
9. **Supplementary Table 9:** Country-wise distribution of the 42 retracted papers published in *Naunyn-Schmiedeberg’s Archives of Pharmacology*.

| Year | Number of Publication |
| --- | --- |
| 2001 | 163 |
| 2002 | 150 |
| 2003 | 160 |
| 2004 | 137 |
| 2005 | 94 |
| 2006 | 94 |
| 2007 | 79 |
| 2008 | 134 |
| 2009 | 123 |
| 2010 | 104 |
| 2011 | 116 |
| 2012 | 116 |
| 2013 | 113 |
| 2014 | 136 |
| 2015 | 118 |
| 2016 | 127 |
| 2017 | 128 |
| 2018 | 130 |
| 2019 | 142 |
| 2020 | 229 |
| 2021 | 223 |
| 2022 | 125 |
| 2023 | 305 |
| 2024 | 909 |

**Supplementary Table 1:** Annual number of publications (all document types) in *Naunyn-Schmiedeberg’s Archives of Pharmacology*, 2001–2024.

| S# | Country | Documents | Citations | S# | Country | Documents | Citations |
| --- | --- | --- | --- | --- | --- | --- | --- |
| 1 | Libyan Arab Jamahiriya | 1 | 18 | 49 | Israel | 9 | 68 |
| 2 | Cyprus | 1 | 15 | 50 | Algeria | 10 | 216 |
| 3 | Lithuania | 1 | 14 | 51 | Hong Kong | 11 | 281 |
| 4 | Macao | 1 | 9 | 52 | Slovakia | 11 | 122 |
| 5 | Costa Rica | 1 | 8 | 53 | Ecuador | 12 | 55 |
| 6 | Lebanon | 1 | 8 | 54 | Finland | 14 | 249 |
| 7 | Nepal | 1 | 3 | 55 | Norway | 15 | 529 |
| 8 | Paraguay | 1 | 2 | 56 | United Arab Emirates | 15 | 193 |
| 9 | Afghanistan | 1 | 1 | 57 | Denmark | 15 | 179 |
| 10 | Armenia | 1 | 1 | 58 | Russian Federation | 16 | 99 |
| 11 | Sudan | 1 | 1 | 59 | Jordan | 16 | 47 |
| 12 | Ethiopia | 1 | 0 | 60 | oman | 17 | 335 |
| 13 | Kyrgyzstan | 1 | 0 | 61 | Romania | 18 | 210 |
| 14 | Qatar | 1 | 0 | 62 | Thailand | 20 | 532 |
| 15 | Rwanda | 1 | 0 | 63 | Argentina | 24 | 211 |
| 16 | Syrian Arab Republic | 1 | 0 | 64 | Belgium | 27 | 702 |
| 17 | Tunisia | 2 | 57 | 65 | Portugal | 30 | 849 |
| 18 | Latvia | 2 | 54 | 66 | Czech Republic | 35 | 568 |
| 19 | Venezuela | 2 | 52 | 67 | Switzerland | 38 | 1024 |
| 20 | Cameroon | 2 | 33 | 68 | Austria | 39 | 889 |
| 21 | Colombia | 2 | 31 | 69 | Malaysia | 39 | 527 |
| 22 | Brunei Darussalam | 2 | 28 | 70 | Mexico | 41 | 595 |
| 23 | Belarus | 2 | 25 | 71 | Iraq | 42 | 436 |
| 24 | Ghana | 2 | 18 | 72 | Hungary | 43 | 967 |
| 25 | Bosnia and Herzegovina | 2 | 7 | 73 | Sweden | 44 | 1356 |
| 26 | Libya | 2 | 2 | 74 | Nigeria | 48 | 508 |
| 27 | Palestine | 2 | 2 | 75 | Pakistan | 51 | 305 |
| 28 | Yemen | 2 | 2 | 76 | Canada | 85 | 1367 |
| 29 | Viet Nam | 2 | 1 | 77 | Taiwan | 86 | 1349 |
| 30 | Slovenia | 3 | 59 | 78 | Netherlands | 91 | 3213 |
| 31 | Indonesia | 3 | 18 | 79 | Spain | 95 | 1930 |
| 32 | Uzbekistan | 3 | 18 | 80 | South Korea | 99 | 1225 |
| 33 | Kazakhstan | 3 | 1 | 81 | France | 107 | 2411 |
| 34 | Uganda | 4 | 56 | 82 | Australia | 114 | 2166 |
| 35 | Serbia | 4 | 38 | 83 | Saudi Arabia | 115 | 1034 |
| 36 | Bulgaria | 4 | 35 | 84 | Poland | 123 | 3083 |
| 37 | Chile | 4 | 25 | 85 | United Kingdom | 150 | 3934 |
| 38 | New Zealand | 4 | 23 | 86 | Italy | 154 | 3957 |
| 39 | Bangladesh | 4 | 19 | 87 | Turkey | 169 | 1836 |
| 40 | Singapore | 4 | 14 | 88 | Brazil | 240 | 4160 |
| 41 | Ireland | 5 | 47 | 89 | Japan | 265 | 5589 |
| 42 | Croatia | 6 | 140 | 90 | Iran | 278 | 2560 |
| 43 | Kuwait | 6 | 65 | 91 | Egypt | 310 | 4396 |
| 44 | Estonia | 6 | 40 | 92 | United States | 320 | 7266 |
| 45 | South Africa | 6 | 39 | 93 | India | 336 | 4051 |
| 46 | Greece | 8 | 107 | 94 | China | 581 | 6128 |
| 47 | Ukraine | 8 | 65 | 95 | Germany | 769 | 17569 |
| 48 | Morocco | 8 | 14 |  |  |  |  |

**Supplementary Table 2:** List of 95 countries with publication and citation counts (all document types) in *Naunyn-Schmiedeberg’s Archives of Pharmacology*, 2001–2024.

| Year | Number of Publication |
| --- | --- |
| 2001 | 160 |
| 2002 | 141 |
| 2003 | 153 |
| 2004 | 123 |
| 2005 | 79 |
| 2006 | 66 |
| 2007 | 64 |
| 2008 | 83 |
| 2009 | 112 |
| 2010 | 93 |
| 2011 | 97 |
| 2012 | 104 |
| 2013 | 95 |
| 2014 | 113 |
| 2015 | 95 |
| 2016 | 113 |
| 2017 | 114 |
| 2018 | 114 |
| 2019 | 122 |
| 2020 | 204 |
| 2021 | 173 |
| 2022 | 86 |
| 2023 | 217 |
| 2024 | 665 |

**Supplementary Table 3:** Annual number of original research articles published in *Naunyn-Schmiedeberg’s Archives of Pharmacology*, 2001–2024.

| S# | Country | Documents | Citations | S# | Country | Documents | Citations |
| --- | --- | --- | --- | --- | --- | --- | --- |
| 1 | armenia | 1 | 1 | 44 | united arab emirates | 7 | 135 |
| 2 | brunei darussalam | 1 | 10 | 45 | slovakia | 9 | 91 |
| 3 | cameroon | 1 | 11 | 46 | denmark | 12 | 173 |
| 4 | colombia | 1 | 14 | 47 | norway | 13 | 508 |
| 5 | costa rica | 1 | 8 | 48 | finland | 14 | 249 |
| 6 | cyprus | 1 | 15 | 49 | jordan | 14 | 43 |
| 7 | ecuador | 1 | 4 | 50 | oman | 15 | 320 |
| 8 | ethiopia | 1 | 0 | 51 | russian federation | 15 | 95 |
| 9 | indonesia | 1 | 0 | 52 | thailand | 16 | 506 |
| 10 | kazakhstan | 1 | 1 | 53 | iraq | 17 | 97 |
| 11 | kyrgyzstan | 1 | 0 | 54 | argentina | 23 | 207 |
| 12 | lithuania | 1 | 14 | 55 | malaysia | 23 | 358 |
| 13 | macao | 1 | 9 | 56 | portugal | 23 | 578 |
| 14 | paraguay | 1 | 2 | 57 | austria | 24 | 564 |
| 15 | rwanda | 1 | 0 | 58 | belgium | 26 | 632 |
| 16 | tunisia | 1 | 4 | 59 | pakistan | 29 | 172 |
| 17 | yemen | 1 | 1 | 60 | mexico | 31 | 370 |
| 18 | belarus | 2 | 25 | 61 | czech republic | 32 | 534 |
| 19 | bosnia and herzegovina | 2 | 7 | 62 | switzerland | 36 | 995 |
| 20 | chile | 2 | 17 | 63 | nigeria | 38 | 322 |
| 21 | ghana | 2 | 18 | 64 | sweden | 41 | 1027 |
| 22 | ireland | 2 | 17 | 65 | hungary | 42 | 925 |
| 23 | latvia | 2 | 54 | 66 | netherlands | 55 | 1596 |
| 24 | new zealand | 2 | 18 | 67 | canada | 70 | 996 |
| 25 | palestine | 2 | 2 | 68 | australia | 74 | 1594 |
| 26 | slovenia | 2 | 28 | 69 | saudi arabia | 74 | 743 |
| 27 | venezuela | 2 | 52 | 70 | south korea | 78 | 1037 |
| 28 | libya | 2 | 20 | 71 | taiwan | 84 | 1277 |
| 29 | bulgaria | 3 | 30 | 72 | spain | 89 | 1630 |
| 30 | serbia | 3 | 21 | 73 | france | 97 | 2186 |
| 31 | singapore | 3 | 12 | 74 | poland | 104 | 2243 |
| 32 | uganda | 3 | 4 | 75 | united kingdom | 118 | 2947 |
| 33 | bangladesh | 4 | 20 | 76 | italy | 136 | 3727 |
| 34 | south africa | 4 | 5 | 77 | turkey | 149 | 1684 |
| 35 | croatia | 5 | 128 | 78 | india | 190 | 2659 |
| 36 | greece | 6 | 100 | 79 | iran | 207 | 1752 |
| 37 | kuwait | 6 | 65 | 80 | brazil | 220 | 3895 |
| 38 | romania | 6 | 121 | 81 | japan | 227 | 4001 |
| 39 | algeria | 7 | 159 | 82 | united states | 231 | 4278 |
| 40 | hong kong | 7 | 229 | 83 | egypt | 242 | 3661 |
| 41 | israel | 7 | 58 | 84 | china | 490 | 5514 |
| 42 | morocco | 7 | 10 | 85 | germany | 594 | 11952 |
| 43 | ukraine | 7 | 34 |  |  |  |  |

**Supplementary Table 4:** List of 85 countries with publication and citation counts for original research articles in *Naunyn-Schmiedeberg’s Archives of Pharmacology*, 2001–2024.

| Year | Number of Publication |
| --- | --- |
| 2001 | 1 |
| 2002 | 5 |
| 2003 | 1 |
| 2004 | 10 |
| 2005 | 12 |
| 2006 | 20 |
| 2007 | 13 |
| 2008 | 13 |
| 2009 | 6 |
| 2010 | 8 |
| 2011 | 18 |
| 2012 | 8 |
| 2013 | 11 |
| 2014 | 11 |
| 2015 | 16 |
| 2016 | 6 |
| 2017 | 5 |
| 2018 | 7 |
| 2019 | 9 |
| 2020 | 9 |
| 2021 | 21 |
| 2022 | 29 |
| 2023 | 62 |
| 2024 | 204 |

**Supplementary Table 5:** Annual number of review articles published in *Naunyn-Schmiedeberg’s Archives of Pharmacology*, 2001–2024.

| S# | Country | Documents | Citations | S# | Country | Documents | Citations |
| --- | --- | --- | --- | --- | --- | --- | --- |
| 1 | Afghanistan | 1 | 1 | 39 | Switzerland | 2 | 29 |
| 2 | Argentina | 1 | 4 | 40 | Thailand | 2 | 27 |
| 3 | Belgium | 1 | 70 | 41 | Viet Nam | 2 | 1 |
| 4 | Brunei Darussalam | 1 | 19 | 42 | Algeria | 3 | 58 |
| 5 | Bulgaria | 1 | 5 | 43 | Hong Kong | 3 | 52 |
| 6 | Cameroon | 1 | 22 | 44 | Ireland | 3 | 30 |
| 7 | Colombia | 1 | 17 | 45 | Uzbekistan | 3 | 18 |
| 8 | Croatia | 1 | 12 | 46 | Portugal | 4 | 266 |
| 9 | Greece | 1 | 6 | 47 | Estonia | 5 | 41 |
| 10 | Hungary | 1 | 44 | 48 | Spain | 5 | 300 |
| 11 | Israel | 1 | 9 | 49 | France | 6 | 210 |
| 12 | Lebanon | 1 | 8 | 50 | United Arab Emirates | 7 | 53 |
| 13 | Libya | 1 | 0 | 51 | Canada | 8 | 261 |
| 14 | Morocco | 1 | 4 | 52 | Japan | 8 | 443 |
| 15 | Nepal | 1 | 4 | 53 | Nigeria | 9 | 186 |
| 16 | New Zealand | 1 | 0 | 54 | Pakistan | 9 | 40 |
| 17 | Norway | 1 | 21 | 55 | Mexico | 10 | 226 |
| 18 | Qatar | 1 | 0 | 56 | Ecuador | 11 | 51 |
| 19 | Russian Federation | 1 | 4 | 57 | Italy | 11 | 214 |
| 20 | Serbia | 1 | 17 | 58 | Poland | 11 | 732 |
| 21 | Singapore | 1 | 2 | 59 | Romania | 12 | 90 |
| 22 | Slovakia | 1 | 31 | 60 | Austria | 13 | 326 |
| 23 | Slovenia | 1 | 31 | 61 | South Korea | 13 | 106 |
| 24 | Sudan | 1 | 1 | 62 | Turkey | 13 | 87 |
| 25 | Syrian Arab Republic | 1 | 0 | 63 | Malaysia | 14 | 165 |
| 26 | Tunisia | 1 | 53 | 64 | Brazil | 16 | 269 |
| 27 | Uganda | 1 | 52 | 65 | Iraq | 22 | 343 |
| 28 | Ukraine | 1 | 31 | 66 | Netherlands | 23 | 1308 |
| 29 | Yemen | 1 | 1 | 67 | United Kingdom | 23 | 840 |
| 30 | Chile | 2 | 8 | 68 | Australia | 29 | 540 |
| 31 | Czech Republic | 2 | 34 | 69 | Saudi Arabia | 34 | 271 |
| 32 | Denmark | 2 | 2 | 70 | Egypt | 40 | 546 |
| 33 | Indonesia | 2 | 19 | 71 | China | 47 | 326 |
| 34 | Jordan | 2 | 5 | 72 | Iran | 60 | 739 |
| 35 | Kazakhstan | 2 | 0 | 73 | United States | 64 | 2000 |
| 36 | Oman | 2 | 15 | 74 | Germany | 121 | 5314 |
| 37 | South Africa | 2 | 34 | 75 | India | 130 | 1388 |
| 38 | Sweden | 2 | 332 |  |  |  |  |

**Supplementary Table 6:** List of 75 countries with publication and citation counts for review articles in *Naunyn-Schmiedeberg’s Archives of Pharmacology*, 2001–2024.

| S# | Authors | Title | Year | Volume | Issue | Page start | Page end | DOI |
| --- | --- | --- | --- | --- | --- | --- | --- | --- |
| 1 | Bibi T.; Khan A.; Khan A.U.; Shal B.; Ali H.; Seo E.K.; Khan S. | Magnolol prevented brain injury through the modulation of Nrf2-dependent oxidative stress and apoptosis in PLP-induced mouse model of multiple sclerosis | 2022 | 395 | 6 | 717 | 733 | 10.1007/s00210-022-02230-6 |
| 2 | Maroufi N.F.; Vahedian V.; Mazrakhondi S.A.M.; Kooti W.; Khiavy H.A.; Bazzaz R.; Ramezani F.; Pirouzpanah S.M.; Ghorbani M.; Akbarzadeh M.; Hajipour H.; Ghanbarzadeh S.; Sabzichi M. | Sensitization of MDA-MBA231 breast cancer cell to docetaxel by myricetin loaded into biocompatible lipid nanoparticles via sub-G1 cell cycle arrest mechanism | 2020 | 393 | 1 | 1 | 11 | 10.1007/s00210-019-01692-5 |
| 3 | Kawadkar M.; Mandloi A.S.; Singh N.; Mukharjee R.; Dhote V.V. | Combination therapy for cerebral ischemia: do progesterone and noscapine provide better neuroprotection than either alone in the treatment? | 2022 | 395 | 2 | 167 | 185 | 10.1007/s00210-021-02187-y |
| 4 | Wahdan S.A.; Tadros M.G.; Khalifa A.E. | Antioxidant and antiapoptotic actions of selegiline protect against 3-NP-induced neurotoxicity in rats | 2017 | 390 | 9 | 905 | 917 | 10.1007/s00210-017-1392-1 |
| 5 | Mahmoud M.F.; Hamdan D.I.; Wink M.; El-Shazly A.M. | Hepatoprotective effect of limonin, a natural limonoid from the seed of Citrus aurantium var. bigaradia, on D-galactosamine-induced liver injury in rats | 2014 | 387 | 3 | 251 | 261 | 10.1007/s00210-013-0937-1 |
| 6 | Naveed M.; Khan S.Z.; Zeeshan S.; Khan A.; Shal B.; Atiq A.; Ali H.; Ullah R.; Zia-ur-Rehman; Khan S. | A new cationic palladium(II) dithiocarbamate exhibits anti-inflammatory, analgesic, and antipyretic activities through inhibition of inflammatory mediators in in vivo models | 2019 | 392 | 8 | 961 | 977 | 10.1007/s00210-019-01645-y |
| 7 | Meng X.; Yuan Y.; Shen F.; Li C. | Heme oxygenase-1 ameliorates hypoxia/reoxygenation via suppressing apoptosis and enhancing autophagy and cell proliferation though Sirt3 signaling pathway in H9c2 cells | 2019 | 392 | 2 | 189 | 198 | 10.1007/s00210-018-1575-4 |
| 8 | Wang C.; Chang R.; Gao G.; Liu X.; Zhang Y. | Fibrauretine reduces ischemia/reperfusion injury via RISK/eNOS activation | 2020 | 393 | 8 | 1515 | 1525 | 10.1007/s00210-019-01770-8 |
| 9 | Wang L.; Wang J.; Zhao H.; Jiang G.; Feng X.; Sui W.; Liu H. | Soyasapogenol B exhibits anti-growth and anti-metastatic activities in clear cell renal cell carcinoma | 2019 | 392 | 5 | 551 | 563 | 10.1007/s00210-018-01607-w |
| 10 | Mohammadpour Y.H.; Khodayar M.J.; Khorsandi L.; Kalantar H. | Betaine alleviates doxorubicin-related cardiotoxicity via suppressing oxidative stress and inflammation via the NLRP3/SIRT1 pathway | 2024 | 397 | 12 | 9981 | 9990 | 10.1007/s00210-024-03261-x |
| 11 | Elseweidy M.M.; Ali S.I.; Shaheen M.A.; Abdelghafour A.M.; Hammad S.K. | Enhancement of cardiac angiogenesis in a myocardial infarction rat model using selenium alone and in combination with PTXF: the role of Akt/HIF-1α signaling pathway | 2024 | 397 | 7 | 4677 | 4692 | 10.1007/s00210-023-02904-9 |
| 12 | Liu Y.; Bi T.; Dai W.; Wang G.; Qian L.; Shen G.; Gao Q. | Lupeol enhances inhibitory effect of 5-fluorouracil on human gastric carcinoma cells | 2016 | 389 | 5 | 477 | 484 | 10.1007/s00210-016-1221-y |
| 13 | Wang Z.; Gao J.; Teng H.; Peng J. | RETRACTED ARTICLE: Role of aminolevulinic acid synthase 1 in doxorubicin-induced oxidative stress to the ardiomyocyte | 2020 | 393 | 11 | 2231 |  | 10.1007/s00210-019-01799-9 |
| 14 | Khan A.U.; Khan A.; Khan A.; Shal B.; Aziz A.; Ahmed M.N.; Islam S.U.; Ali H.; Shehzad A.; Khan S. | Inhibition of NF-κB signaling and HSP70/HSP90 proteins by newly synthesized hydrazide derivatives in arthritis model | 2021 | 394 | 7 | 1497 | 1519 | 10.1007/s00210-021-02075-5 |
| 15 | Alafifi S.A.; Wahdan S.A.; Elhemiely A.A.; Elsherbiny D.A.; Azab S.S. | Modulatory effect of liraglutide on doxorubicin-induced testicular toxicity and behavioral abnormalities in rats: role of testicular-brain axis | 2023 | 396 | 11 | 2987 | 3005 | 10.1007/s00210-023-02504-7 |
| 16 | AL-Eitan L.; Kharmah H.A. | Effect of EMB-FUBINACA on brain endothelial cell angiogenesis: Expression analysis of angiogenic markers | 2025 | 398 | 2 | 1613 | 1624 | 10.1007/s00210-024-03322-1 |
| 17 | Jiang Y.; Hong D.; Lou Z.; Tu X.; Jin L. | Lupeol inhibits migration and invasion of colorectal cancer cells by suppressing RhoA-ROCK1 signaling pathway | 2020 | 393 | 11 | 2185 | 2196 | 10.1007/s00210-020-01815-3 |
| 18 | Alzahrani S.; Ajwah S.M.; Alsharif S.Y.; Said E.; El-Sherbiny M.; Zaitone S.A.; Al-Shabrawey M.; Elsherbiny N.M. | Isoliquiritigenin downregulates miR-195 and attenuates oxidative stress and inflammation in STZ-induced retinal injury | 2020 | 393 | 12 | 2375 | 2385 | 10.1007/s00210-020-01948-5 |
| 19 | Kamran G.; Kharl H.A.A.; Malik M.N.H.; Younis W.; Nadeem H.; Zubair A.M.; Malik M.A.H.; Jahan S.; Ahmed I.; Shabbir R.; Akram A.; Anjum I.; Atif M.; Raza M.; Kamla G.Z. | Novel mannich-based derivative of 2-mercaptobenzimidazole (AK7): a new candidate for the treatment of inflammatory arthritis owing to its NF-κB1 inhibitory potential | 2023 | 396 | 4 | 811 | 827 | 10.1007/s00210-022-02359-4 |
| 20 | Elseweidy M.M.; Mahrous M.; Ali S.I.; Shaheen M.A.; Younis N.N. | Vitamin D alleviates cognitive dysfunction and brain damage induced by copper sulfate intake in experimental rats: focus on its combination with donepezil | 2023 | 396 | 9 | 1931 | 1942 | 10.1007/s00210-023-02449-x |
| 21 | Sun Y.; Lv B.; Zhang X. | RETRACTED ARTICLE: Knock-down of LncRNA-XIST induced glioma cell death and inhibited tumorigenesis by regulating miR-137/SLC1A5 axis-mediated ROS production | 2021 | 394 | 3 | 557 |  | 10.1007/s00210-020-01831-3 |
| 22 | Mitra S.S.; Ghorai M.; Nandy S.; Mukherjee N.; Kumar M.; Radha; Ghosh A.; Jha N.K.; Proćków J.; Dey A. | Barbaloin: an amazing chemical from the ‘wonder plant’ with multidimensional pharmacological attributes | 2022 | 395 | 12 | 1525 | 1536 | 10.1007/s00210-022-02294-4 |
| 23 | Wahdan S.A.; Azab S.S.; Elsherbiny D.A.; El-Demerdash E. | Piceatannol protects against cisplatin nephrotoxicity via activation of Nrf2/HO-1 pathway and hindering NF-κB inflammatory cascade | 2019 | 392 | 11 | 1331 | 1345 | 10.1007/s00210-019-01673-8 |
| 24 | Yan W.; Wu J.; Song B.; Luo Q.; Xu Y. | Treatment with a brain-selective prodrug of 17β-estradiol improves cognitive function in Alzheimer’s disease mice by regulating klf5-NF-κB pathway | 2019 | 392 | 7 | 879 | 886 | 10.1007/s00210-019-01639-w |
| 25 | Khan A.; Khan A.; Shal B.; Aziz A.; Ahmad S.; Amin M.U.; Ahmed M.N.; Zia-ur-Rehman; Khan S. | Ameliorative effect of two structurally divergent hydrazide derivatives against DSS-induced colitis by targeting Nrf2 and NF-κB signaling in mice | 2022 | 395 | 10 | 1167 | 1188 | 10.1007/s00210-022-02272-w |
| 26 | Haddadi R.; Eyvari-Brooshghalan S.; Nayebi A.M.; Sabahi M.; Ahmadi S.A. | Neuronal degeneration and oxidative stress in the SNc of 6-OHDA intoxicated rats; improving role of silymarin long-term treatment | 2020 | 393 | 12 | 2427 | 2437 | 10.1007/s00210-020-01954-7 |
| 27 | Elsayed A.A.; Menze E.T.; Tadros M.G.; Ibrahim B.M.M.; Sabri N.A.; Khalifa A.E. | Effects of genistein on pentylenetetrazole-induced behavioral and neurochemical deficits in ovariectomized rats | 2018 | 391 | 1 | 27 | 36 | 10.1007/s00210-017-1435-7 |
| 28 | Corrie L.; Singh H.; Gulati M.; Vishwas S.; Chellappan D.K.; Gupta G.; Paiva-Santos A.C.; Veiga F.; Alotaibi F.; Alam A.; Eri R.D.; Prasher P.; Adams J.; Paudel K.R.; Dua K.; Singh S.K. | Polysaccharide-fecal microbiota-based colon-targeted self-nanoemulsifying drug delivery system of curcumin for treating polycystic ovarian syndrome | 2024 | 397 | 9 | 6721 | 6743 | 10.1007/s00210-024-03029-3 |
| 29 | Erdogan M.A.; Yılmaz O.A. | Rottlerin and genistein inhibit neuroblastoma cell proliferation and invasion through EF2K suppression and related protein pathways | 2023 | 396 | 10 | 2481 | 2500 | 10.1007/s00210-023-02473-x |
| 30 | Attri S.; Kumar A.; Kaur K.; Kaur P.; Punj S.; Bedi N.; Tuli H.S.; Arora S. | Assessment of anti-psoriatic activity of bakuchiol-loaded solid lipid nanoparticles-based gel: design, characterization, and mechanistic insight via NF-kB signaling pathway | 2023 | 396 | 9 | 2105 | 2125 | 10.1007/s00210-023-02445-1 |
| 31 | Wang Q.-L.; Wang L.; Li Q.-Y.; Li H.-Y.; Lin L.; Wei D.; Xu J.-Y.; Luo X.-J. | Micafungin exerts antitumor effect on breast cancer and osteosarcoma through preventing EMT in tumor cells in an USP7/AKT/GSK-3β pathway-dependent manner | 2024 | 397 | 6 | 4447 | 4459 | 10.1007/s00210-023-02903-w |
| 32 | El-Demerdash A.A.; Menze E.T.; Esmat A.; Tadros M.G.; Elsherbiny D.A. | Protective and therapeutic effects of the flavonoid “pinocembrin” in indomethacin-induced acute gastric ulcer in rats: impact of anti-oxidant, anti-inflammatory, and anti-apoptotic mechanisms | 2021 | 394 | 7 | 1411 | 1424 | 10.1007/s00210-021-02067-5 |
| 33 | Yin J.; Yin Q.; Liang B.; Mi R.; Ai H.; Chen L.; Wei X. | Chrysophanol suppresses growth and metastasis of T cell acute lymphoblastic leukemia via miR-9/PD-L1 axis | 2020 | 393 | 2 | 273 | 286 | 10.1007/s00210-019-01778-0 |
| 34 | Liu Y.; Bi T.; Wang G.; Dai W.; Wu G.; Qian L.; Gao Q.; Shen G. | Lupeol inhibits proliferation and induces apoptosis of human pancreatic cancer PCNA-1 cells through AKT/ERK pathways | 2015 | 388 | 3 | 295 | 304 | 10.1007/s00210-014-1071-4 |
| 35 | Rashid S.A.; Naseem F.; Shah P.A.; Hashmi H.B.; Mazher M.; Mubarak M.S.; Sharifi-Rad J.; Badar M. | Development and evaluation of methotrexate-loaded nanoemulsion formulation for topical treatment of psoriasis | 2025 | 398 | 2 | 1765 | 1783 | 10.1007/s00210-024-03364-5 |
| 36 | Zhang J.; Wang Q.; Wang Q.; Guo P.; Wang Y.; Xing Y.; Zhang M.; Liu F.; Zeng Q. | Chrysophanol exhibits anti-cancer activities in lung cancer cell through regulating ROS/HIF-1a/VEGF signaling pathway | 2020 | 393 | 3 | 469 | 480 | 10.1007/s00210-019-01746-8 |
| 37 | Xue Y.; Sun C.; Hao Q.; Cheng J. | Astaxanthin ameliorates cardiomyocyte apoptosis after coronary microembolization by inhibiting oxidative stress via Nrf2/HO-1 pathway in rats | 2019 | 392 | 3 | 341 | 348 | 10.1007/s00210-018-1595-0 |
| 38 | Abdel-Hamid N.M.; ElNakeeb N.A.; El-Senduny F.F. | Efficient chemosensitizing and antimetastatic combinations of a naturally occurring trans-ferulic acid with different chemotherapies on an in vitro hepatocellular carcinoma model | 2023 | 396 | 8 | 1741 | 1747 | 10.1007/s00210-023-02431-7 |
| 39 | Semerci Sevimli T.; Sevimli M.; Ghorbani A.; Şahintürk V.; Qomi Ekenel E.; Ertem T.; Demir Cevizlidere B.; Altuğ B.; Tomsuk Ö.; Uysal O.; Güneş Bağış S.; Avci H.; Çemrek F.; Ahmadova Z. | The analysis of boric acid effect on epithelial-mesenchymal transition of CD133 + CD117 + lung cancer stem cells | 2024 | 397 | 9 | 6791 | 6802 | 10.1007/s00210-024-03062-2 |
| 40 | Mounier N.M.; Wahdan S.A.; Gad A.M.; Azab S.S. | Role of inflammatory, oxidative, and ER stress signaling in the neuroprotective effect of atorvastatin against doxorubicin-induced cognitive impairment in rats | 2021 | 394 | 7 | 1537 | 1551 | 10.1007/s00210-021-02081-7 |
| 41 | Li Q.; Jiang C.; Wang Y.; Wei M.; Zheng H.; Xu Y.; Xu X.; Jia F.; Liu K.; Sun G.; Zang J.; Mo P. | Resibufogenin suppresses tumor growth and inhibits glycolysis in ovarian cancer by modulating PIM1 | 2019 | 392 | 12 | 1477 | 1489 | 10.1007/s00210-019-01687-2 |
| 42 | Liu Y.; Bi T.; Liu L.; Gao Q.; Shen G.; Qin L. | S-Adenosylmethionine synergistically enhances the antitumor effect of gemcitabine against pancreatic cancer through JAK2/STAT3 pathway | 2019 | 392 | 5 | 615 | 622 | 10.1007/s00210-019-01617-2 |

**Supplementary Table 7:** List of 42 retracted papers published in *Naunyn-Schmiedeberg’s Archives of Pharmacology*. This data was updated and retrieved in June 2025.

| S# | Affiliation | Number of Publications |
| --- | --- | --- |
| 1 | Ain Shams University, Cairo, Egypt. | 6 |
| 2 | Faculty of Pharmacy - Ain Shams University, Cairo, Egypt. | 6 |
| 4 | Zagazig University, Zagazig, Egypt | 5 |
| 3 | Quaid-i-Azam University, Islamabad, Pakistan | 4 |
| 5 | Wujiang No. 1 People's Hospital, Suzhou, China | 2 |
| 6 | Mansoura University, Mansoura city, Egypt. | 2 |
| 8 | Tabriz University of Medical Sciences, Iran | 2 |
| 9 | University of Azad Jammu and Kashmir, Kashmir | 2 |
| 10 | Port Said University, Port Said, Egypt. | 2 |
| 11 | Uttaranchal University, Uttarakhand, India | 2 |
| 12 | Abasyn University, Peshawar, Pakistan | 2 |

**Supplementary Table 8:** Institutions with at least two publications among the 42 retracted papers in *Naunyn-Schmiedeberg’s Archives of Pharmacology*. This data was updated and retrieved in June 2025.

| S# | Country | Number of Publications |
| --- | --- | --- |
| 1 | China | 15 |
| 2 | Egypt | 11 |
| 3 | Pakistan | 6 |
| 4 | India | 4 |
| 5 | Iran | 3 |
| 6 | Australia | 2 |
| 7 | Jordan | 2 |
| 8 | Saudi Arabia | 2 |
| 9 | South Korea | 2 |
| 10 | Turkey | 2 |
| 11 | Canada | 1 |
| 12 | Ecuador | 1 |
| 13 | Germany | 1 |
| 14 | Malaysia | 1 |
| 15 | Mexico | 1 |
| 16 | Poland | 1 |
| 17 | Portugal | 1 |
| 18 | Türkiye | 1 |
| 19 | United Arab Emirates | 1 |
| 20 | United States | 1 |

**Supplementary Table 9:** Country-wise distribution of the 42 retracted papers published in *Naunyn-Schmiedeberg’s Archives of Pharmacology*. This data was updated and retrieved in June 2025.
