# Supplementary material for: Country-specific citation disparities in Naunyn–Schmiedeberg’s Archives of Pharmacology from 2001 to 2024
Source: Naunyn Schmiedebergs Arch Pharmacol. 2025 Aug 14;399(2):2113–28. doi: 10.1007/s00210-025-04499-9 (PMC12901205; doi:10.1007/s00210-025-04499-9)
Supplement: Supplementary file 2 — (DOCX 216 KB) [file 210_2025_4499_MOESM2_ESM.docx]

**Legends**

**Supplementary Figure 1:** Linear regression analysis of countries with fewer than 10 research publications (all document types). The x-axis represents the number of publications, and the y-axis shows the total number of citations. Data includes all document types published in Naunyn-Schmiedeberg’s Archives of Pharmacology (NSAP) from 2001 to 2024.

**Supplementary Figure 2:** Linear regression analysis of countries with fewer than 10 research publications (only research articles). The x-axis represents the number of publications, and the y-axis shows the total number of citations. Data includes all document types published in Naunyn-Schmiedeberg’s Archives of Pharmacology (NSAP) from 2001 to 2024.

**Supplementary Figure 3:** Linear regression analysis of countries with fewer than 10 research publications (only reviews). The x-axis represents the number of publications, and the y-axis shows the total number of citations. Data includes all document types published in Naunyn-Schmiedeberg’s Archives of Pharmacology (NSAP) from 2001 to 2024.

**
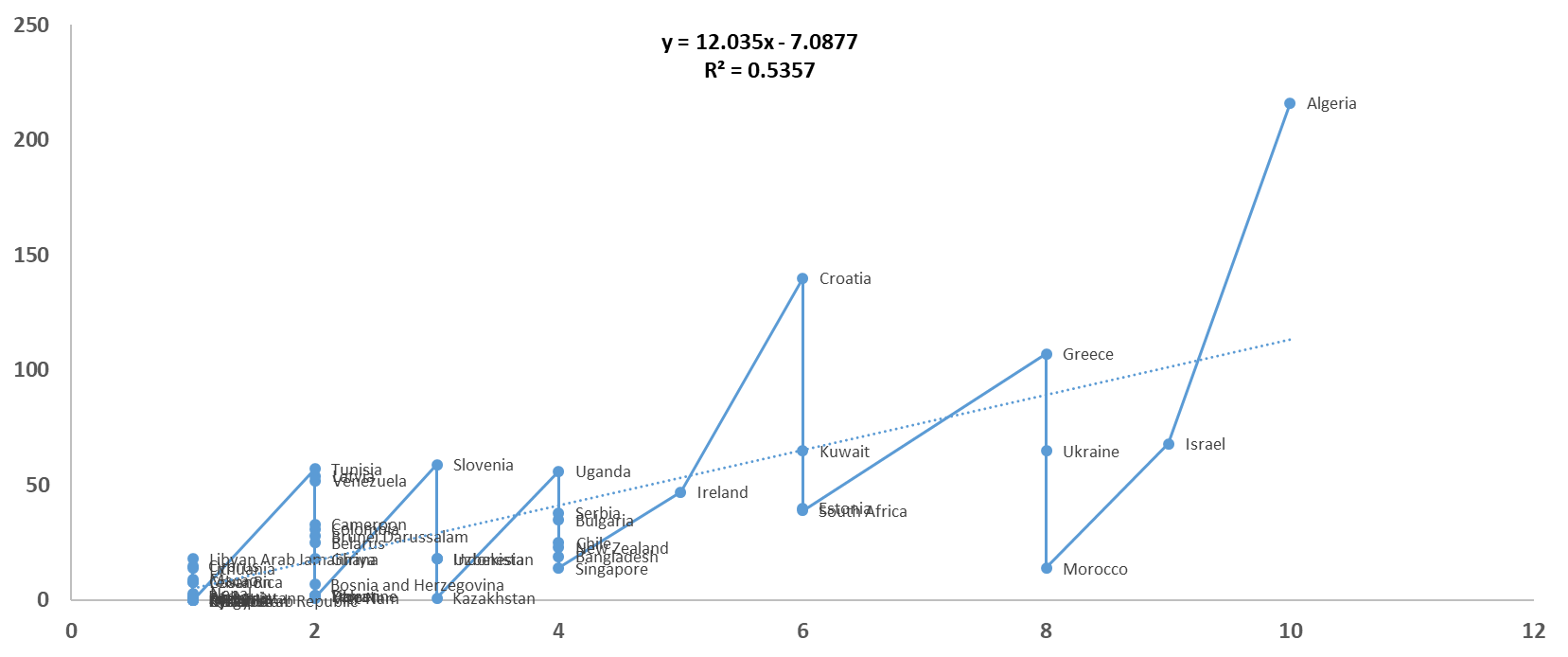
**

**Supplementary Figure 1:** Linear regression analysis of countries with fewer than 10 research publications (all document types). The x-axis represents the number of publications, and the y-axis shows the total number of citations. Data includes all document types published in Naunyn-Schmiedeberg’s Archives of Pharmacology (NSAP) from 2001 to 2024.

**
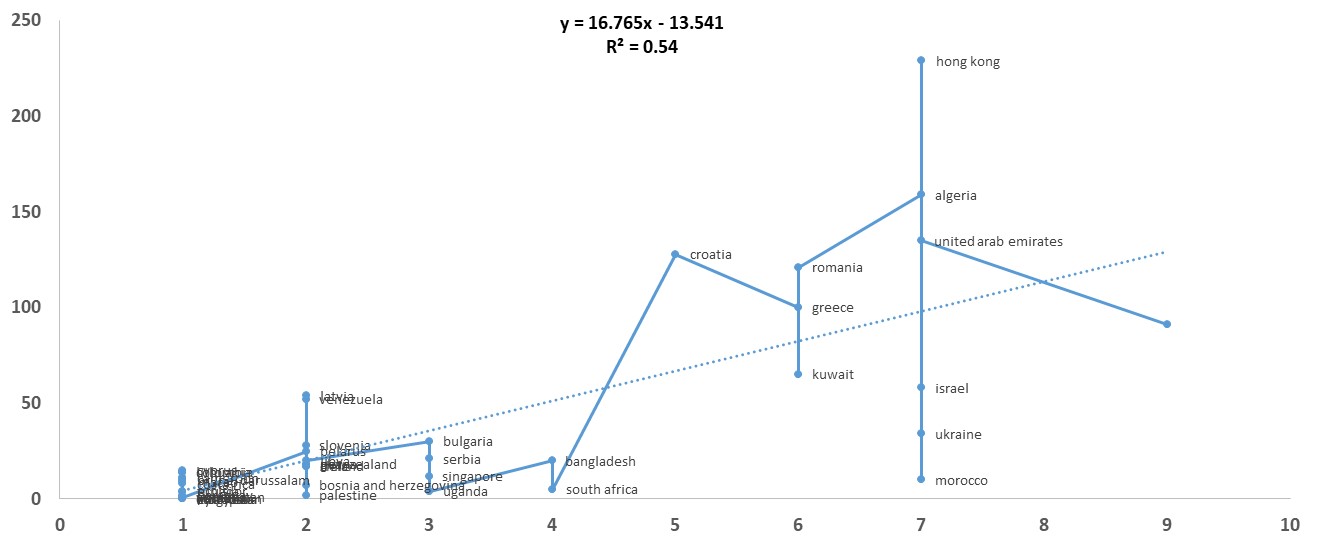
**

**Supplementary Figure 2:** Linear regression analysis of countries with fewer than 10 research publications (only research articles). The x-axis represents the number of publications, and the y-axis shows the total number of citations. Data includes all document types published in Naunyn-Schmiedeberg’s Archives of Pharmacology (NSAP) from 2001 to 2024.

**
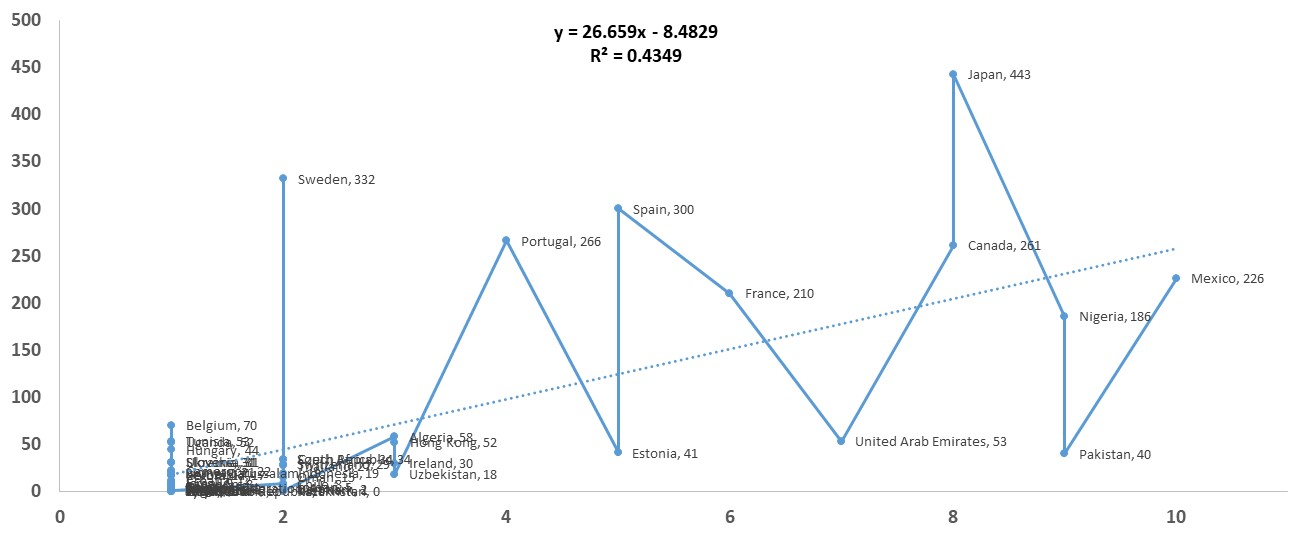
**

**Supplementary Figure 3:** Linear regression analysis of countries with fewer than 10 research publications (only reviews). The x-axis represents the number of publications, and the y-axis shows the total number of citations. Data includes all document types published in Naunyn-Schmiedeberg’s Archives of Pharmacology (NSAP) from 2001 to 2024.
